# Effects of Land Cover on the Movement of Frugivorous Birds in a Heterogeneous Landscape

**DOI:** 10.1371/journal.pone.0156688

**Published:** 2016-06-03

**Authors:** Natalia Stefanini Da Silveira, Bernardo Brandão S. Niebuhr, Renata de Lara Muylaert, Milton Cezar Ribeiro, Marco Aurélio Pizo

**Affiliations:** 1 Departament of Ecology, Spatial Ecology and Conservation Lab, Universidade Estadual Paulista Julio de Mesquita Filho, Rio Claro, São Paulo, Brazil; 2 Departament of Zoology, Universidade Estadual Paulista Julio de Mesquita Filho, Rio Claro, São Paulo, Brazil; Auburn University, UNITED STATES

## Abstract

Movement is a key spatiotemporal process that enables interactions between animals and other elements of nature. The understanding of animal trajectories and the mechanisms that influence them at the landscape level can yield insight into ecological processes and potential solutions to specific ecological problems. Based upon optimal foraging models and empirical evidence, we hypothesized that movement by thrushes is highly tortuous (low average movement speeds and homogeneous distribution of turning angles) inside forests, moderately tortuous in urban areas, which present intermediary levels of resources, and minimally tortuous (high movement speeds and turning angles next to 0 radians) in open matrix types (e.g., crops and pasture). We used data on the trajectories of two common thrush species (*Turdus rufiventris* and *Turdus leucomelas*) collected by radio telemetry in a fragmented region in Brazil. Using a maximum likelihood model selection approach we fit four probability distribution models to average speed data, considering short-tailed, long-tailed, and scale-free distributions (to represent different regimes of movement variation), and one distribution to relative angle data. Models included land cover type and distance from forest-matrix edges as explanatory variables. Speed was greater farther away from forest edges and increased faster inside forest habitat compared to urban and open matrices. However, turning angle was not influenced by land cover. Thrushes presented a very tortuous trajectory, with many displacements followed by turns near 180 degrees. Thrush trajectories resembled habitat and edge dependent, tortuous random walks, with a well-defined movement scale inside each land cover type. Although thrushes are habitat generalists, they showed a greater preference for forest edges, and thus may be considered edge specialists. Our results reinforce the importance of studying animal movement patterns in order to understand ecological processes such as seed dispersal in fragmented areas, where the percentage of remaining habitat is dwindling.

## Introduction

Human-caused landscape modifications such as habitat loss, fragmentation, and land use degradation have negative impacts on biodiversity and ecosystem functions [1, 2, 3]. Besides having direct effects on individuals and ecological interactions, these modifications may also affect animal movement, a process that links individual behavior to biotic interactions. Thus, studying how animal movement may responds to such alterations can improve our understanding of population and community ecology in a changing world [4–5].

Movement features may mediate predation, mortality, and resource encounter rates in altered environments [6–7], comprising one of the key processes in the maintenance of animal populations in fragmented landscapes [8]. The interaction among animal morphology, behavior and landscape structure produces different movement signatures [9–10], each of which distinctly influence animals’ responses to human-caused modifications. For instance, forest-specialist species, which rely mainly on forest resources, tend to have their home range inside forested areas and avoid leaving forest fragments. On the other hand, habitat generalist species that use a wider range of resources are expected to be less restricted in space, using a variety of habitat types including those subject to intense human influence [6, 11, 12]. Comprehending how habitat generalist species move in fragmented areas is especially important to understand complex interactions, such as animal-mediated seed dispersal. For example, the movement of frugivorous birds may influence not only their own population connectivity but also plant population dynamics [13, 14, 15, 16, 17].

A landscape is typically composed of several landscape elements. One can usually classify the landscape using the patch-corridor-matrix model, which considers patches of habitat embedded in matrix and structurally connected by narrow tracts of habitat, the corridors. In scenarios of high fragmentation, the matrix is the most extensive and connected landscape element, playing an important role in the functioning of the entire landscape [18].

Theory predicts that habitat generalist species in heterogeneous landscapes with high-quality, low-risk matrices have high movement probabilities (i.e. a high probability or per capita rate of leaving a current location or habitat patch [19, 20, 21, 22, 23]). Thus, these species may show weak responses to boundaries between two different types of vegetation or habitat patches. In low-risk matrices, habitat generalist species are not expected to avoid a specific vegetation type when near boundaries, moving long distances in such landscapes [6]. Likewise, they tend to perform tortuous paths inside habitat patches, where food resources are abundant, and present straighter trajectories in open matrices such as grasslands or agricultural fields [6, 24, 25, 26, 27]. In the context of anthropogenic landscape changes generally characterized by habitat loss and reduced matrix quality, how animal movement characteristics change is a widely open question. What are the effects of land cover on the movement of common and supposed generalist animals? This second question is particularly important given the tendency of individuals to spend more time in the matrix, potentially facing increased mortality risk and reduced movement success [6]. Several common bird species that are habitat generalists in the extensively modified Atlantic Forest landscapes are suitable models to address such questions.

The presence of habitat-generalist bird species in urban and suburban areas is common worldwide, probably due to their adaptation or low specificity for roosts and food resources (i.e. broad environmental tolerance) [28]. For example, 31% of Brazilian bird species have been recorded in urban areas [29]. Thus, urban and suburban landscapes can provide many resources for common and generalist birds, such as thrushes [30]. Moreover, thrushes may benefit more from suburban landscapes than from pastures, probably due to fewer resources in the latter compared to native forest fragments and urban environments [31].

Forest areas have an abundance of fruit trees and insects on which thrushes feed, and where they are relatively protected from most predators [32, 33, 34, 35]. In such resource-rich environments, animals are expected to perform short displacements and present tortuous trajectories [6, 7, 24, 27]. In contrast, urban areas are constantly changing and suffer direct human influence. Additionally, resource availability may vary according to those changes, then in terms of availability of food resources in urban areas must differ from forest areas. [36, 37, 38].

**Fig 1:**
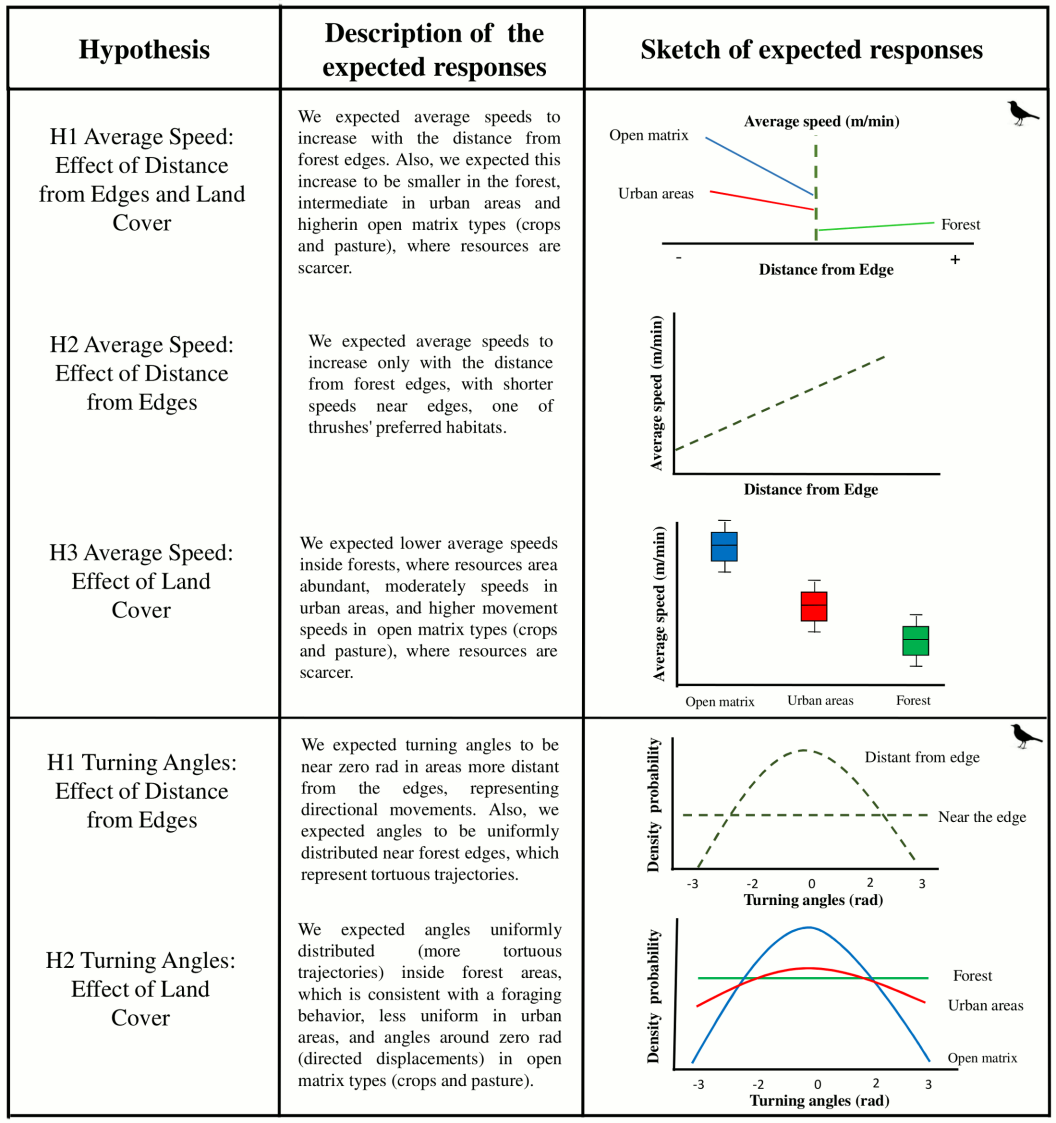
Predictions for average speed and turning angles of thrushes' trajectories in a fragmented landscape. Alternative hypotheses are explained and shown graphically considering the effects of land cover variables, land cover type, and distance to forest edges. In hypothesis H1 for average speed, zero values represent the contact area between two land cover classes (i.e. the edge).

Although urban areas provide food sources, due to the presence of lawns and fruit trees [39, 40, 41], nest predation and young mortality rates can be higher in urban environments and juveniles seek areas of native forest after leaving their nests [32, 33, 34]. Therefore, we hypothesized that movement by thrushes is highly tortuous (low average movement speeds and homogeneous distribution of turning angles) inside forests, moderately tortuous in urban areas, which present intermediary levels of resources, and minimally tortuous (high movement speeds and turning angles next to 0 radians) in open matrix types (e.g., crops and pasture), based upon optimal foraging models [24–25] and empirical evidence [26–27] (Fig 1). Strong responses are represented by extremely high turning angles, suggesting the avoidance of or preference for a certain land cover type. Due to the habitat-generalist habit of thrushes, we did not expect strong responses in relation to forest edges [6]. Because the two thrush species investigated (Tudus leucomelas and Turdus rufiventris) demonstrate some preference for edge areas we also hypothesized that thrushes would exhibit low mean speeds and long periods of time spent near the edges [30–42]. Expected responses of average speed and turning angles with land cover variables are explained and shown graphically in Fig 1.

## Materials and Methods

### Ethic statement and survey permits

We affirm that fieldwork did not involve endangered or protected species. In accordance with the environmental legislation of Brazil (norm N° 154/2007) we received authorization (N° 39790–1, authentication code N° 56882872) from the Brazilian Federal Environmental Agency (Instituto Brasileiro do Meio Ambiente e dos Recursos Naturais Renováveis—IBAMA), for mist netting and tagging with radio transmitters from 06/2013 and 08/2014. For more details, please access the SISBIO (Sistema de Autorização e Informação em Biodiversidade), www.icmbio.gov.br/sisbio. In addition, the study was conducted on private land, in and around Sítio Moinho Velho and located adjacent properties. For further information, please contact one of the owners of Sítio Moinho Velho, Dr. Marco Aurélio Pizo.

### Study area

The study area was located in rural areas of Itatiba, state of São Paulo, southeastern Brazil (22° 57'S, 46° 44'W). The region has a temperate tropical climate, with temperatures between 18°C and 25°C (annual average: 20.6°C) and a mountainous terrain formed by the Serra da Jurema ridge. Historically, the region was covered by semi-deciduous Atlantic rainforest [43], which was fragmented centuries ago to make room for pasture and agricultural production [31]. The Atlantic Forest is a highly fragmented tropical biome composed mainly of large tracts of pasture and agricultural crops as well as small forest fragments covering less than 16% of its original extent. It is also the most urbanized region in Brazil [44–45].

**Fig 2:**
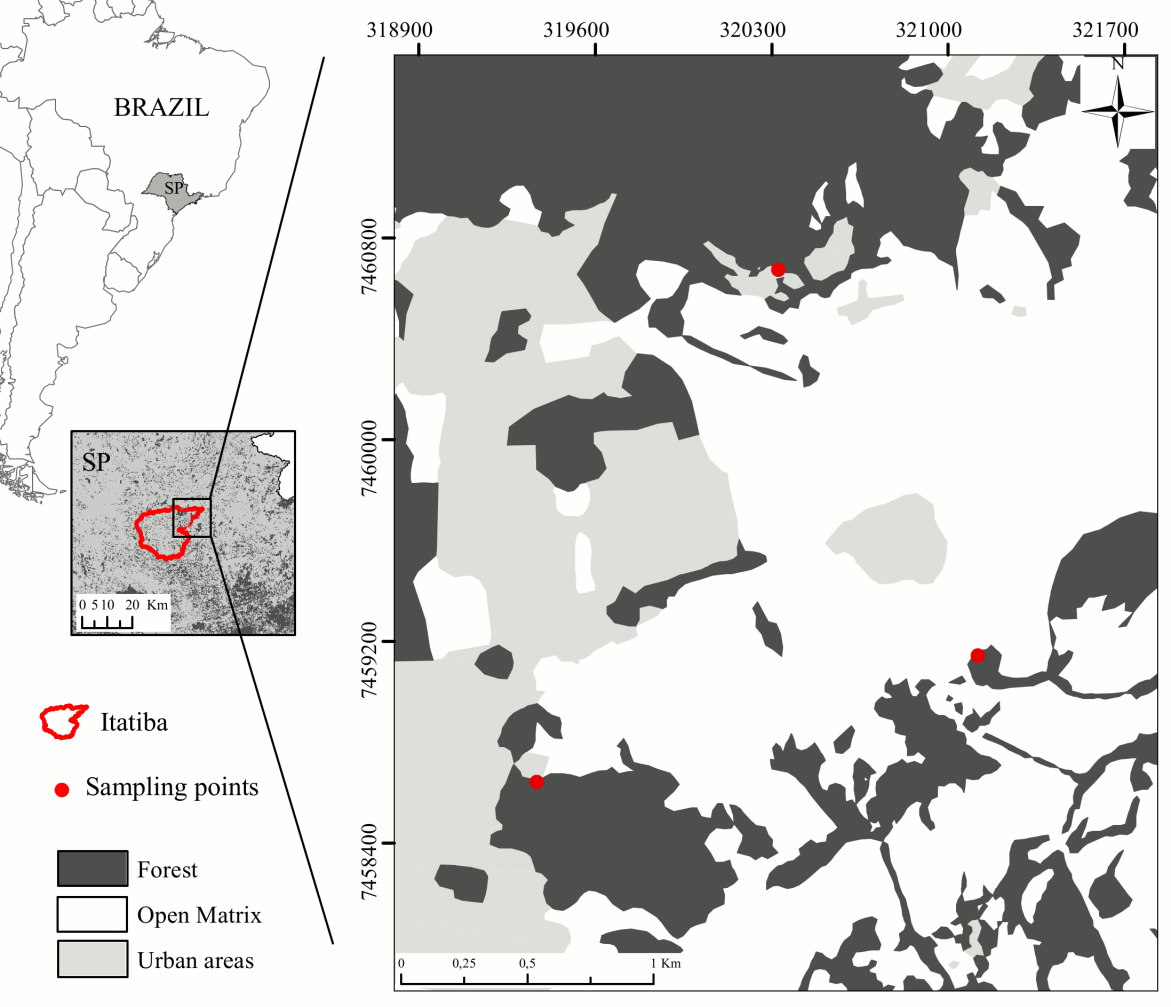
Study area encompasses the city limits of Itatiba, State of São Paulo, southeastern Brazil. The red points correspond to locations where *T*. *rufiventris* and *T*. *leucomelas* were captured. Land cover composition was: 51.0% pasture, 0.1% crops (mainly *Citrus* and corn fields), 14.9% urban areas, 1.1% water, 23.4% forest, 9.5% old plantations of *Eucalyptus* embedded in forested areas. Live fences are formed by lines of native trees, such as *Casearia sylvestris*, *Lithraea mollioides* and *Erythroxyllum deciduum*, located on different properties.

The study area is dominated by three distinct land cover types: a) forest fragments in different successional stages (fragment areas varying from 1 to 20 ha, some of which are completely isolated while others are connected by elongated forest corridors), mixed with old plantations of *Eucalyptus*, with a native understory (hereafter, forest), b) urban areas, consisting of residential condominiums, farms, and ranches, and c) open matrix, that consist on pasture, crops, and are traversed by a few natural hedges (live fences; 4 to 12 m width, 150 to 400 m length) formed by lines of native trees, particularly species that produce small fleshy fruits (<5 mm in diameter) dispersed by birds such as *Caseariasy lvestris*, *Lithrae amollioides* and *Erythroxylum deciduum* [46] (Fig 2). The natural hedges are primarily composed of native plants and were here also considered as forest habitat type (S1 Fig). Land cover composition of the entire study area included 51% pasture, 0.1% crops (mainly *Citrus* and corn fields), 14.9% urban areas, 1.1% water, 23.4% forest fragments, and 9.5% old plantations of *Eucalyptus* embedded in forested areas. We considered all non-forest cover types in the study area as potentially adequate habitat for the studied species due to their supposed tendency to use the space outside forests, especially during reproductive periods when fewer animals were observed in native forests [35].

To understand the influence of landscape on thrush movement, we classified the areas according to the most abundant land cover classes (forest, urban areas, and open matrices), but also in binary land cover classes (forest and non-forest). These two classifications allow us to explore whether thrushes recognize and respond to different landscape elements or if the typical binary classification is sufficient to capture the variation in their movement.

### Radiotelemetry

Fieldwork was done from June 2013 to June 2014. We focused on the species *Turdus rufiventris* and *Turdus leucomelas* (S1 Table), which were captured with mist nets positioned in the edge of selected fragments at three sites within the study area (Fig 2). A research permit granted by the Chico Mendes Institute for Conservation and Biodiversity (SISBIO research license N° 39790–1) supported the fieldwork activities.

Radio transmitters (model A1080, ATS Advanced Telemetry Systems) were affixed to each captured individual as a backpack and weighed 1.9 g, less than 5% of the body weight of the animal [47] (see S1 Table for individual body weights). Altogether, we monitored ten individuals: five *T*. *leucomelas* (two females and three males) and five *T*. *rufiventris* (two females and three males).

We marked locations for individuals through triangulation of the bearings obtained by two teams of observers positioned at GPS-mapped stations (Model Garmin 60CSX, error up to 10 m in open field). We tried to collect location positions approximately every 20 min throughout a tracking session. We also observed locations recorded directly from visual contact with the tagged individuals. Given the occasional loss of radio signal due to the thrushes’ high mobility, and the use of visual contact data, bird trajectories had an irregular distribution of time among relocations. The landscape was mountainous, which made difficult the triangulation of the bearings among forest fragments. Moreover, transmitters were small, what can decrease the distance range of the signal. Therefore, many daily points were eventually lost because of difficulty to move around and capture the signal in this type of relief. We completed 180 days of sampling, which permitted us to gather 336 points of activity for the ten monitored individuals. The collected points of activity were found mostly near the edges of forested areas (S2 Fig).

### Data analysis

The general protocol for animal movement studies is based on recording movement paths from direct observation or telemetry. The movement paths are analyzed as discretized trajectories, which can be represented as a series of linear displacements between location fixes (step lengths) separated by angular shifts (turning angles) [48, 49, 50]. In some cases, movement rates or average speeds are reported instead of step lengths in fixed time intervals [49, 50, 51, 52]. Here we described thrush movement by their average speeds (in m/min) and relative turning angles (in radians), the response variables of this study. To consider speeds in a biologically meaningful way, we excluded time ranges longer than two hours and intervals in which there was no data collection, such as during night. We corroborated this exclusion by applying the Pearson's correlation test to verify the relationship between time and speed within a range of two points. We observed small negative correlation between speeds and intervals (r = -0.18; t = -3.07; df = 273, p = 0.002); high speeds occurred only over short intervals. After excluding these locations, samples sizes were 275 for average speeds and 242 for turning angles (S1 Table).

Robust methods such as state-space models [53–54] and step selection functions [55–56] have been developed to analyze movement patterns. These methods require a continuous series of movement data, such as those recorded with GPS equipment or radio-tracking methods. However, small and highly mobile bird species such as *Turdus* thrushes are difficult to track continuously and accurately. Methods less dependent on abundant and continuous data have been used to assess land cover effects on movement [57–58]. These methods separate the analysis into two parts: one to identify the scale and distribution of movement rates and turning angles, and another to estimate the effects of landscape structure on movement patterns. Here we used a maximum likelihood model selection approach to identify the probability distribution (among short-tailed, long-tailed, and scale-free distributions) and the combination of covariates–land cover and distance to forest edges–that best explain variation in movement speed and turning angles of thrushes.

Four probability distributions were used to fit average speeds: exponential, Weibull, Lévy, and truncated Lévy distributions. Exponential distributions are short-tailed and consider that animals perform trajectories within a characteristic spatial scale (individual displacement lengths are centered around a typical value) [59], so that their path resembles a Brownian motion with normal diffusion properties [25]. On the other hand, Lévy distributions are long-tailed power-law distributions with infinite variance, giving rise to super diffusive dynamics. They have scale-free properties, which means that they allow values for displacement lengths and speeds much greater than the mean values of the distribution. In practice, they can be represented by clusters of short displacements with high tortuosity separated by some very long moves. Truncated Lévy (or bounded Pareto) distributions are modified versions which limit the maximum value of the distribution but still maintain a long-tail and super diffusion properties over relatively large time extensions [25]. Weibull distributions are more flexible and can present short- or heavy-tails, or even a non-zero mode, depending on their parameters, which can represent differences in behavior or responses to environmental gradients. We believe that by considering these four families of probability distributions we could represent distinct combinations of movement patterns to be fit to the data.

**Fig 3:**
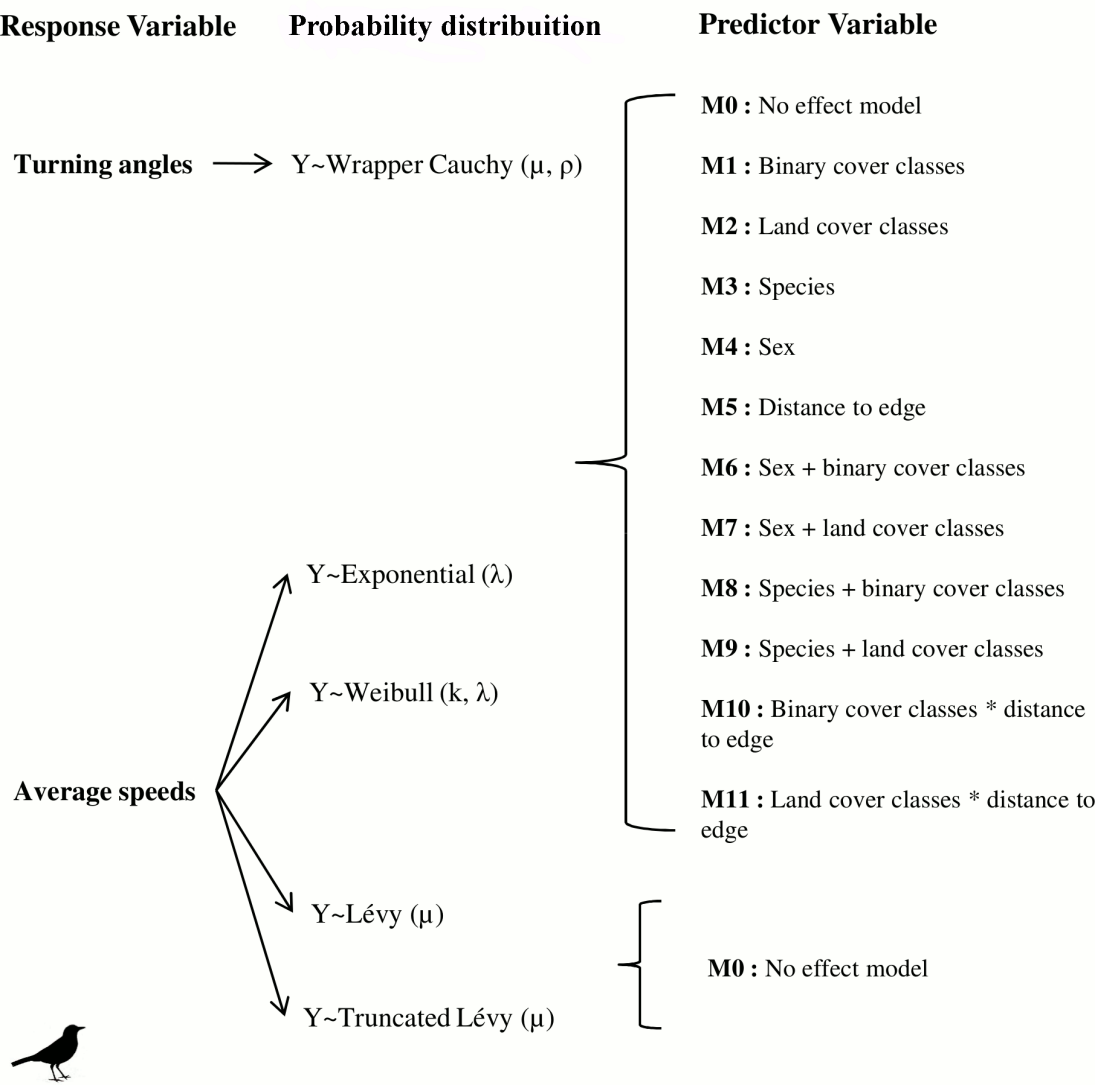
Set of distributions and their parameter settings used to explain variation in the response variables. Models used for the model selection approach to understand the contribution of landscape variables, sex, and species to thrush movement. Models M6-M9 considered additive effects of covariates, while models M10 and M11 consider their interaction. Y is the response variable.

To fit turning angles, we used a wrapped Cauchy distribution. Negative log-likelihood functions were built to fit each of these distributions to our data. After model fitting, we used Akaike Information Criterion corrected for small samples (AICc) and Akaike weights (*w*, relative likelihood of the model) to compare competing models [60]. We considered that differences in AIC values less than two (ΔAICc ≤ 2) were equally plausible to explain the observed patterns, but that for models with similar AICc values, we gave priority to simpler models by parsimony. To identify how landscape variables affect movement, we assumed that one of the parameters of these distributions (λ for exponential, k for Weibull, and μ for wrapped Cauchy) was a function that varied depending on the combinations of variables (Fig 3). These models were also compared to no-effect models, where all parameters were constant, and to models considering the effects of sex and species, to make sure the variation in movement variables were indeed due to landscape covariates. For truncated Lévy distributions, only no-effect models were considered, since they inherently consider a greater variation in movement speed or step lengths.

The landscape variables considered were: (i) distance to edge, the distance to the nearest forest edge in absolute terms, considering edge as the transition zone between different land cover types; (ii) binary land cover, which refers to the land cover class of the location where each thrush displacement started, considering a binary classification of forest and non-forest; and (iii) land cover, which is equivalent to (ii) but refers to a three-level classification—forest areas, urban areas, and open matrices—separating the non-forest areas into two classes, urban areas and pasture/plantations. All models, comments on model fitting, and the scripts of model implementation are available in the supplementary material (S1, S2, S3, and S4 Texts). To check for the effects of individual variation and the small sample size for some individuals (S1 Table) on results, we also developed a hierarchical version of the most plausible model for average speeds, considering individuals as a random effect on intercepts. As the results were qualitatively similar, comments on individual variation, description of the model, and scripts are not shown here, but are available in supplementary material (S5 and S6 Texts). All analyses were performed using R 3.1.2 environment (R Dev. Core Team 2014) and bbmle [61] and adehabitat assembly packages [62].

## Results

We recorded 275 displacements and 242 turning angles for the ten individuals of the two thrush species. The number of speed values and turning angles differ due to breaks in the trajectories, such as, when there was an interruption of the radio signal and at long intervals at the night period. The first displacement did not have a relative previous angle to be compared. The most plausible model in explaining the average movement speed was the exponential model (M11), which considered land cover (forest, open matrices, and urban areas) and forest edge distances influencing the pattern of average speeds and had an exponential error (Table 1). Regarding movement speed no other model was as plausible as (AICc difference < 2) or had comparable weight to M11.

**Table 1:** Competing models describing average speed for *Turdus leucomelas* and *T*. *rufiventris*. Plausible model in italic. K is the number of estimated parameters and the *w* is the Akaike weights (relative likelihood of the model).

Response variable	Distribution	Models		dAICc	K	*w*
Average speed	*Exponential*	*M11*	Land cover classes * distance to edge	*0*.*0*	*6*	*0*.*881*
	Exponential	M10	Binary cover classes * distance to edge	4.1	4	0.114
	Weibull	M11	Land cover classes * distance to edge	13.1	7	0.001
	Weibull	M8	Species + binary cover classes	13.3	4	0.001
	Weibull	M9	Species +land cover classes	15.1	5	<0.001
	Weibull	M10	Binary cover classes * distance to edge	15.4	5	<0.001
	Weibull	M3	Species	17.3	3	<0.001
	Exponential	M9	Species + land cover classes	21.2	4	<0.001
	Exponential	M8	Species + binary cover classes	21.5	3	<0.001
	Weibull	M1	Binary cover classes	22.1	3	<0.001
	Weibull	M6	Sex + binary cover classes	22.5	4	<0.001
	Exponential	M5	Distance to edge	22.7	2	<0.001
	Weibull	M2	Land cover classes	24.2	4	<0.001
	Weibull	M5	Distance to edge	24.5	3	<0.001
	Weibull	M7	Sex + land cover classes	24.5	5	<0.001
	Weibull	M0	No effect model	26.0	2	<0.001
	Weibull	M4	Sex	26.2	3	<0.001
	Exponential	M3	Species	29.0	2	<0.001
	Exponential	M6	Sex + binary cover classes	34.3	3	<0.001
	Exponential	M7	Sex+ land cover classes	35.4	4	<0.001
	Exponential	M4	Sex	39.0	2	<0.001
	Exponential	M1	Binary cover classes	41.3	2	<0.001
	Exponential	M2	Land cover classes	42.6	3	<0.001
	Exponential	M0	No effect model	47.1	1	<0.001
	Truncated lévy	M0	No effect model	237.6	1	<0.001
	Lévy	M0	No effect model	319.4	1	<0.001

**Fig 4:**
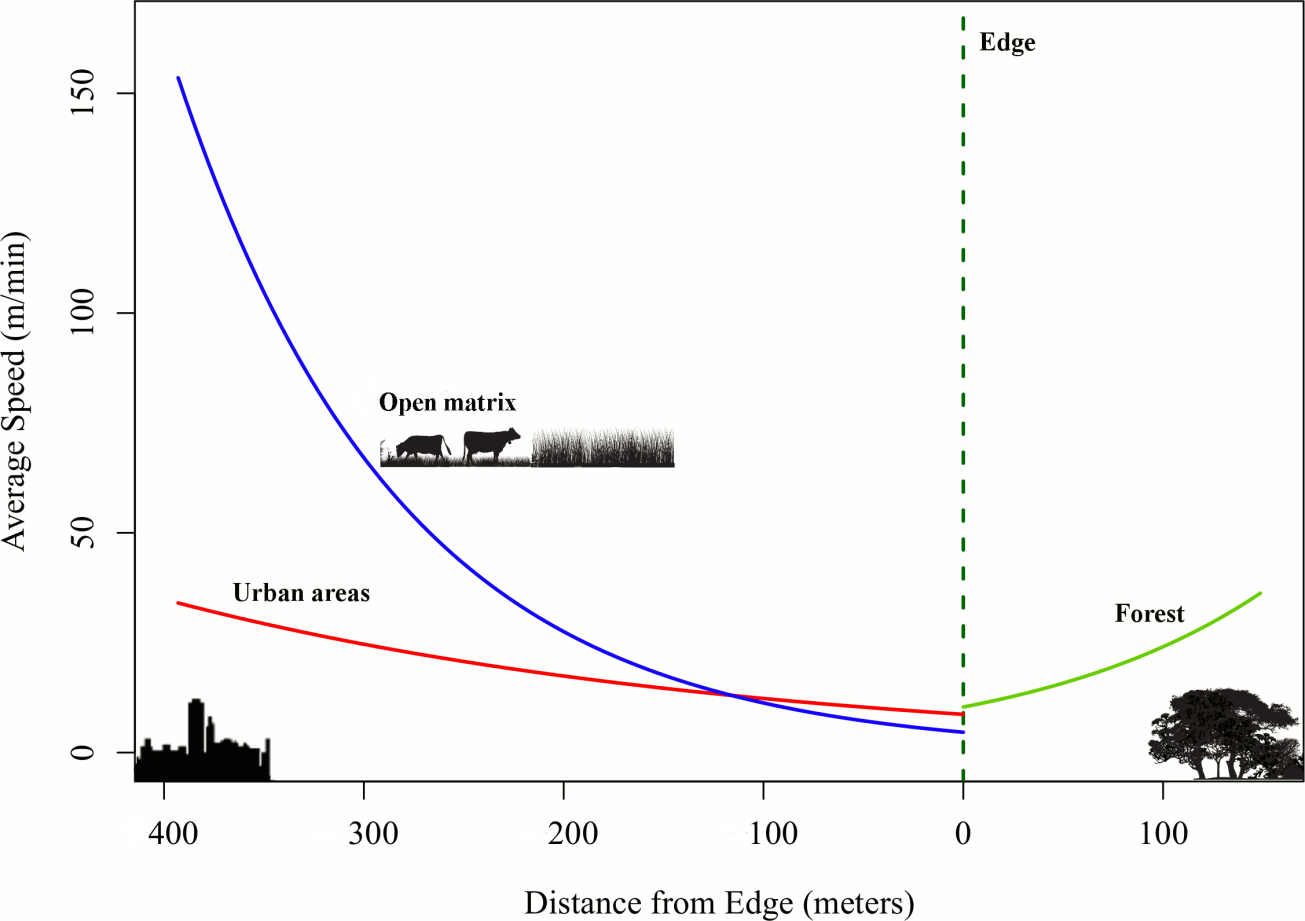
Average speeds of thrushes as a function of distances from edge, considering land cover classes. The dashed line represents the forest edges; green (forest), blue (open matrix—pastures and crops) and red (urban areas) lines represent the expected values for the mean speeds of individuals according to the exponential model M11, which considers the parameter settings being influenced by landscape variables (forest, open matrices and urban areas) and the forest edge distances. Speeds increase as individuals move away from forest edges.

Average speeds increased as thrushes moved away from the forest edges either towards forest interior or matrices (urban and other areas). Both the initial speeds and the rates of speed variation, increased for thrushes moving from the forest edges to the forest interior. Contrary to our expectations, the previous rates were higher than the rates for thrushes moving from the edges towards the matrices (Fig 4). Also, the average speeds were higher in the forest than in other habitats (open matrix and urban areas with approximate values; S3 Fig). Individuals presented some variation in their response, but the qualitative response to landscape variables was similar when this variation was accounted for (S5 Text).

**Fig 5:**
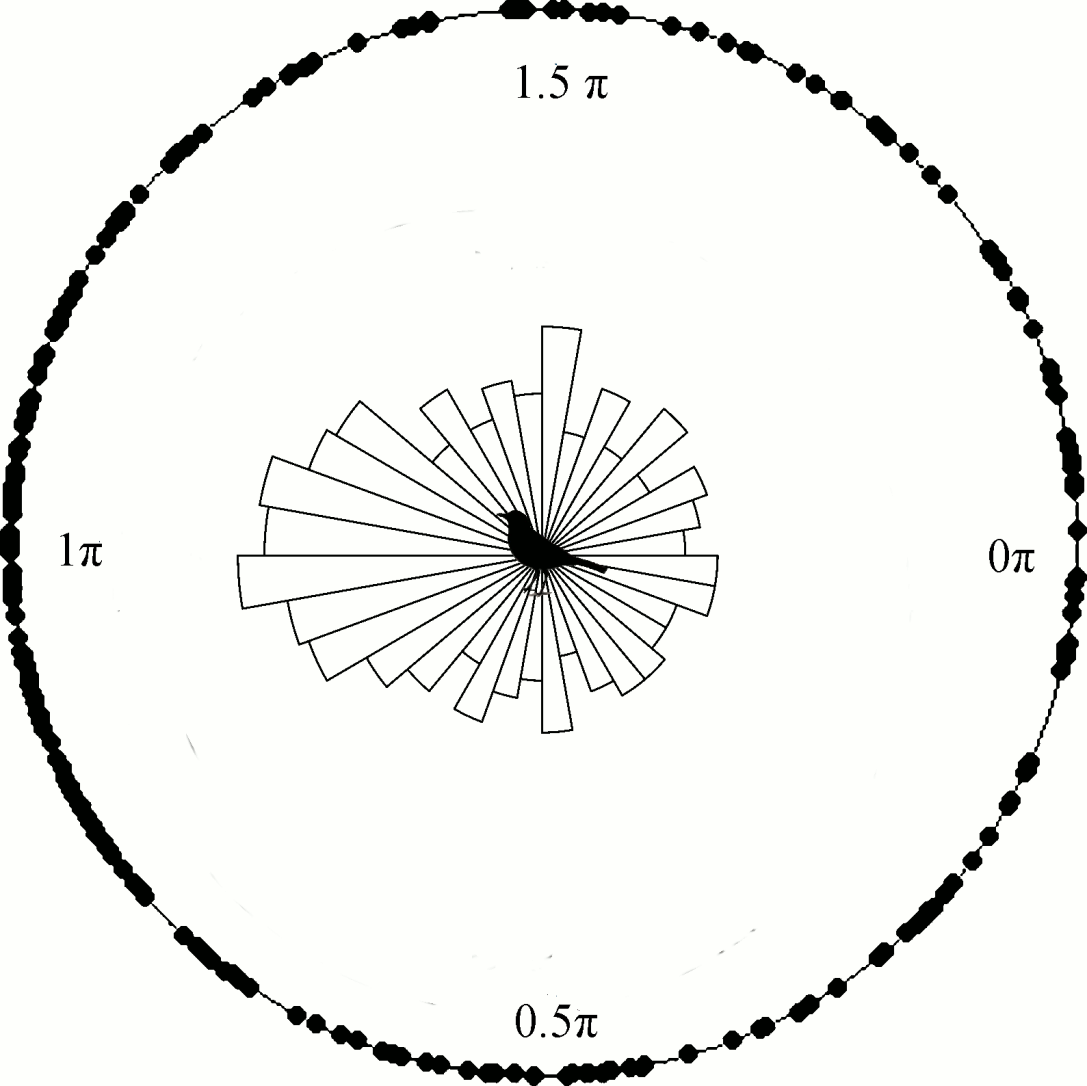
Distribution of turning angles. Black points around the circle represent the relative angle observations. Note the high frequency of angle values of +180° or −180° (π or -π radians), which represents more abrupt turns and is characteristic of tortuous walks.

In the case of turning angle, the model with the absence of effect was equally plausible to the others, but was the most parsimonious (Table 2). This show a weak or unpredictable effects of all selected predictors on turning angles, since both extrinsic (land cover, distance to forest edges) and intrinsic variables (sex, species) explain turning angle variation equally well. The no-effect model shows that high turning angles are common, indicating a very tortuous movement pattern with many twists and relative angles close to 180 degrees, independent of land cover (Fig 5). In summary, trajectories of both thrush species, *T*. *leucomelas* and *T*. *rufiventris*, resembled habitat and edge dependent, tortuous random walks, with a well-defined movement scale inside each land cover type.

**Table 2:** Competing models using turning angles for the trajectories for *Turdus leucomelas* and *T*. *rufiventris*. Plausible model in italic. K is the number of estimated parameters and the *w* Akaike weights (relative likelihood of the model) and the *w* is the Akaike weights (relative likelihood of the model).

Response Variable	Models		dAICc	K	*w*
Turning Angles	M0	*No effect model*	*0*.*0*	*2*	*0*.*333*
	M1	*Binary cover classes*	*1*.*9*	*3*	*0*.*130*
	M3	*Species*	*1*.*9*	*3*	*0*.*129*
	M5	*Distance to edge*	*2*.*0*	*3*	*0*.*122*
	M4	*Sex*	*2*.*0*	*3*	*0*.*120*
	M8	Species + binary cover classes	3.8	4	0.049
	M2	Land cover classes	3.9	4	0.047
	M6	Sex + binary cover classes	3.9	4	0.046
	M10	Binary cover classes * distance to edge	5.9	5	0.017
	M9	Species +land cover classes	6.0	5	0.016
	M7	Sex +land cover classes	6.2	5	0.014
	M11	Land cover classes * distance to edge	10.0	7	0.002

## Discussion

Many variables can affect animals’ movement behavior and how it relates to interactions such as seed dispersal [63]. Intrinsic characteristics of the seed disperser (such as physiology and morphology), combined with extrinsic characteristics, particularly landscape structure and availability of food and shelter, influence the animals foraging mode and seed deposition over space and time [64–65]. In this study, thrush species were shown to perform very tortuous movement paths for all land cover types and landscape characteristics (Table 2, Fig 5). The thrushes’ movement speed changed with land cover, but the birds were able to move freely through the different habitat types in the landscape. These movement characteristics indicate a plastic behavior and, considering thrushes’ ecological role, may translate into a great capacity for seed distribution over long distances among different landscape elements [23, 66, 67]. However, the birds differed in average flight speed among landscape features: forest edges and land cover type (Table 1, Fig 4).

### Thrushes as habitat generalists or edge specialists?

Compared to forest-specialist species, habitat generalist birds exhibit a greater propensity to move across or to use different habitat types without being significantly affected by changes in land cover. Forest-specialist species depend on large expanses of forest and high availability of resources [33]. However, being a habitat generalist does not imply a lack of habitat preferences. Instead, the habitat generalist is able to successfully modulate aspects of behavior in different habitat types. For example, juveniles of *Turdus assimilis* in Costa Rica had a higher survival rate in forested areas when compared to pastures and agricultural habitats, which may indicate a threshold amount of native forest providing critical resources for the persistence of the population [32]. Here we found low average speeds and a high frequency of observations near forest edges (Fig 4, S2 Fig), as well as a tendency to move faster far from such edges, although the thrushes used forest, open matrix and urban environments. This may represent an edge preference by *T*. *leucomelas* and *T*. *rufiventris*, together with the use different habitats. Therefore, they might be neither habitat generalists nor edge specialists only, but fall intermediate to both classifications. As in the present study, thrushes of the species *T*. *rufiventris* moved slowly near forest edges, but also spent considerable time in forest edges and were highly adapted to urbanized regions in others studies [34–35]. Both cover types—forest edges and urban areas—may consist of foraging sites and, therefore, birds would pass quickly through forest interior regions to reach the edge areas. We also found very tortuous trajectories, with turning angles near 180°. This may represent “round trips” away and back to forest edges, reinforcing their tendency to stay next to them.

Thrushes of the genus *Turdus* are similar in several aspects, including feeding habits and morphology [30–42]. This similarity translates to movement behavior, and may explain why our models that considered differences between species poorly explained observed trajectories, reinforcing the hypothesis that different species of the genus can be ecologically similar in terms of movement patterns.

Using the same focal species, Vogel et al. [68] detected no interspecific differences in the use of habitat and forest strata in relation to the interior and edge of forest fragments, despite morphological differences, which corroborates our findings. However, when considering a broader assemblage, birds of the same genus (e.g. *Turdus*) may present different responses to landscape attributes in terms of movement [69]. When aspects of thrush behavior are combined to simulate seed dispersal, they may present complementarity in the seed dispersal process [69], which has implications for the quantification of seed dispersal components. The difference between this study and ours may lie in the fact that the authors measured resource abundance itself, while we used land cover type as a proxy for resource and roost abundance. Our results support that the two studied species have similar movement patterns and consequently may exhibit similar roles as seed dispersers. Also, the tendency to stay next and move slower near forest edges may increase the probability of seed deposition by the studied thrush species in this micro habitat, what deserves further investigation. Still, the extent to which the similarity in movement showed here applies to other thrush species is still unknown, indicating a need to evaluate in other species.

### Land cover types, resource distribution and movement

Variability in thrush movement speed was best explained by an exponential distribution model and the expected value for speeds varied with land cover type. This pattern consisted of a characteristic scale of steps, lengths, and speeds for each land cover type, such that a thrush’s trajectory can be represented as a multi-scaled random walk in which the scale of speeds depends on habitat type [10, 25, 70]. Exponential signatures for animal movement have been observed in nomadic animals, which cover large areas in search of resources and do not settle in one single area [24–71]. The exponential signatures for movement was also observed in the present study for both species (maximum displacement = 1643.9 m/10 min, and minimum displacement = 2.82 m/15 min) and may relate to the availability of resources. This behavior, comprising steps with a well defined scale in places of high resource availability, has also been observed in other animals such as albatrosses (*Diomedea exulans*) [26], deer (*Dama dama*), and bees (*Bombus terricola*) [72]. As observed here, the scale of movement depends on habitat (and resource abundance), which is expected from theoretical models [70–73]. In fact, a theoretical models were tested with mud snails (*Hydrobia ulvae*), whose movement patterns responded to experimentally manipulated food availability [10]. However, the mud snail movements indicated that it is often hard to differentiate the evidence of a combination of exponential distributions and of Lévy signatures. In the present study we did found evidence for exponential distributions instead of a Lévy distribution of average speeds, although this result may have been affected by methodological issues–thrushes (and therefore radio tags) are small, the radio signal is spatially limited, and the landscape terrain is rough, which turn difficult to record very long and fast displacements, characteristics of Lévy walks and flights [25].

We expected low average speeds in forests, followed by intermediate speeds in urban areas and higher speeds in pastures. Surprisingly, the results showed the opposite relationship, with a higher mean speed within the area containing the largest forest patch (223 ha) and a lower average speed in areas containing large amounts of other habitats. These other habitats were mainly formed by pastures and urbanized areas, which comprised 51% and 15% of the total study area, respectively. This unexpected pattern can be related to the foraging habits. Thrushes may also use common routes inside forest patches, especially when they have large home ranges, and tend to move quickly to forest edges, so that the speed inside forest is high. Thrushes have also been observed using living fences (per. obs. by NSS), narrow tracts of forest-like environments that may function as corridors, inducing fast displacements over forest cover. Interestingly, due to the diversification of the environment, urban areas near forest fragments offer several features such as considerable roost and food resource availability that can benefit adaptable species [29].

A study [46] previously quantified the fruit availability in the hedges of the same study region, which appeared to be seasonal, peaking mostly in the period between September and February, and did not differ between forest patches and hedges. Similarly, no differences were observed between the amount of arthropods in fragments and hedges [46]. This information can also help us understand the slow speeds observed near the sampling point to the South, where pasture predominated and natural hedges connected forest fragments. Furthermore, our personal observations revealing that the secondary forests of the region are poor in fruits may also have contributed to the high speed of thrushes through forest interior, given the influence of vegetation type on the ease or success with which birds obtain resources [74]. Despite harboring many living trees, several secondary forests seem to have equal or fewer food resources compared to orchards near urban settlements [46]. Secondary forests should, therefore, be less attractive to opportunistic frugivores, particularly in terms of foraging aspects and seasonal effects. Mainly during the dry season, thrushes may take advantage of orchards around urban areas, live fences, and isolated trees in pasture or crop fields, due to the presence of fleshy fruits and invertebrates. Thus, the presence of these elements in fragmented areas favor plastic species such as *Turdus* but are less attractive to habitat-specialist species [75]. Also, this region has passed through an unusual drought, coinciding with the sampling period [76], which inhibited fruit production and may have led thrushes to find resources outside the forest, since fruit production was retarded (per. obs. by NSS).

Predation is also a factor to be considered when analyzing animal movement. As common birds, thrushes of genus *Turdus* including *T*. *rufiventris* and *T*. *leucomelas* may commonly display mobbing behavior, facing predators when other birds that are potentially a prey are aware of predation risk [77, 78, 79]. It was beyond our study to attempt tracking response to predation risk, but overall we did not observe any predator attack on thrushes in any of the land cover types (per. obs. by NSS).

The above discussion highlights the importance of noting the variety of search patterns emerging in habitats that contain a differential distribution of resources. Future research should include a spatially explicit model for resource distribution to measure organisms’ foraging trajectories and their possible outcomes, such as the number of seeds spread combined with information on foraging behavior. Investigating thrushes can improve our understanding of forest regeneration, and their role as seed dispersers may become even more perceptible in landscapes with low amounts of forest and in defaunated landscapes—where they are among the most common seed dispersers [15].

## Conclusions

Our study represented the first step in studying the fine scale movement of thrushes in Neotropical fragmented areas. Thrushes seem to be resilient and adapted to several forms of environmental degradation and showed a tendency to be edge specialists. Trajectories of thrushes resemble a highly tortuous habitat-dependent exponential distribution of movement speeds, representing a model of random walk with well-defined scales for each land cover type. These paths seem closely related to the foraging behavior of habitat-generalist animals, which have adapted to different environments, including urbanized areas. This result can deepen the understanding of animal responses to habitat fragmentation. Furthermore, the results can support the management of remaining areas, as these frugivorous birds—potential dispersers of many plant species and among the main seed dispersers in Atlantic Forest [15–31]—move easily over fragmentation gradients and should be considered in the development of management strategies for biodiversity conservation. We also suggest the integration of profiles for common frugivorous species into landscape resilience simulation models, particularly when planning the areas as set-asides for natural regeneration and predicting aspects of forest growth on a large scale [80, 81, 82], which is necessary for the restoration of the Atlantic Forest remnants.

## Supporting Information

S1 Fig Photo of the study area located in the countryside of Itatiba, São Paulo, Brazil. (PDF)

S2 Fig Frequency of activity points as a function of distance to forest edge. (PDF)

S3 Fig Influences of land use classes (a), sex (b) and species (c) on the average speeds of thrushes within fragmented landscapes of southeastern Brazil.
(PDF)

S1 File Full data from two species of thrushes of the genus Turdus sp. collected by using the radio telemetry methodology from June 2013 to June 2014 in the city limits of Itatiba, São Paulo, Brazil. (PDF)

S1 Table Individuals of *Turdus leucomelas* and *Turdus rufiventris* followed by radio telemetry from June 2013 to June 2014 in the city limits of Itatiba, São Paulo. (PDF)

S1 Text Movement analysis R script for thrushes in fragmented landscapes. (PDF)

S2 Text Description of the probability distributions used to explain variation in movement data. (PDF)

S3 Text Description of the models used to fit movement data. (PDF)

S4 Text Details of movement data fit. (PDF)

S5 Text Considerations of individual variation on the effects of land cover on thrush movement. (PDF)

S6 Text Hierarchical model script including individual variation. (PDF)
