# Supplementary material for: Effects of Land Cover on the Movement of Frugivorous Birds in a Heterogeneous Landscape
Source: PLoS One. 2016 Jun 3;11(6):e0156688. doi: 10.1371/journal.pone.0156688 (PMC4892584; doi:10.1371/journal.pone.0156688)
Supplement: S1 Fig — (PDF) [file pone.0156688.s001.pdf]

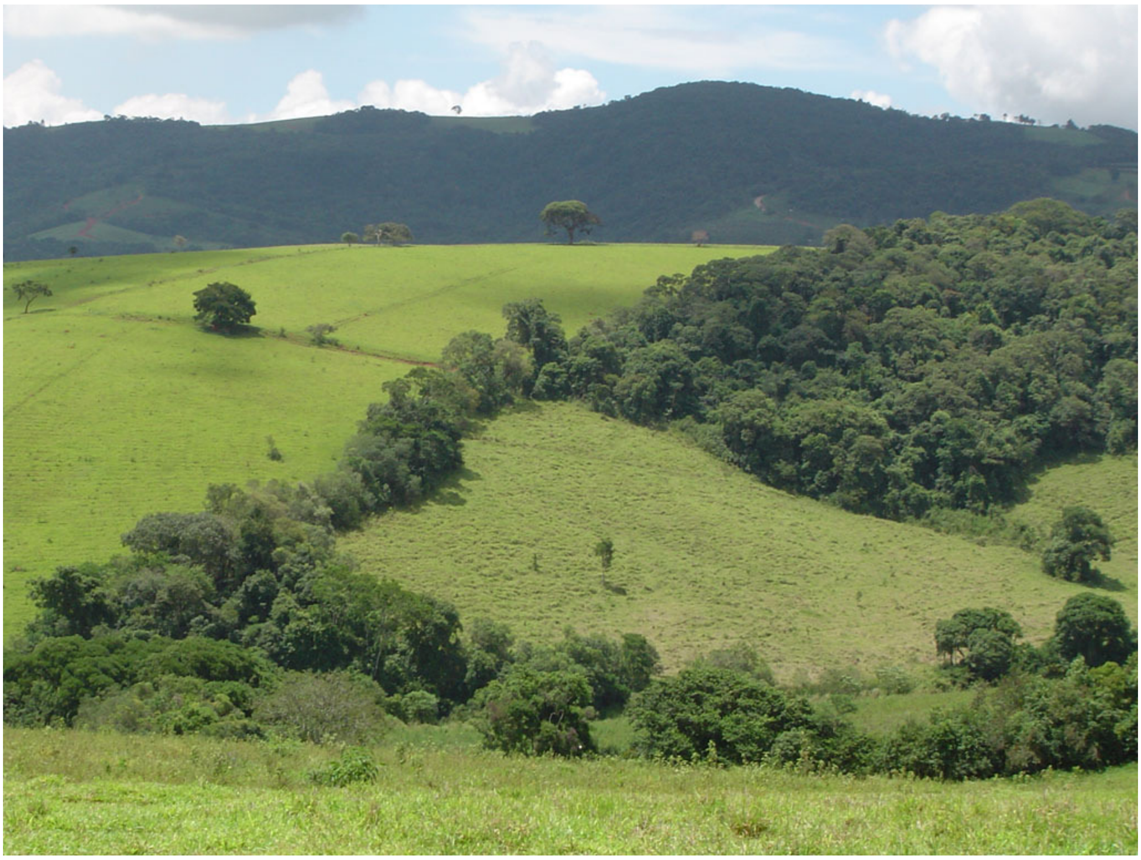

S1 Fig. Photo of the study area located in the countryside of Itatiba, São Paulo, Brazil. Note large pasture areas and the presence of natural hedges connecting forest fragments.
