# Supplementary material for: Effects of Land Cover on the Movement of Frugivorous Birds in a Heterogeneous Landscape
Source: PLoS One. 2016 Jun 3;11(6):e0156688. doi: 10.1371/journal.pone.0156688 (PMC4892584; doi:10.1371/journal.pone.0156688)
Supplement: S2 Fig — (PDF) [file pone.0156688.s002.pdf]

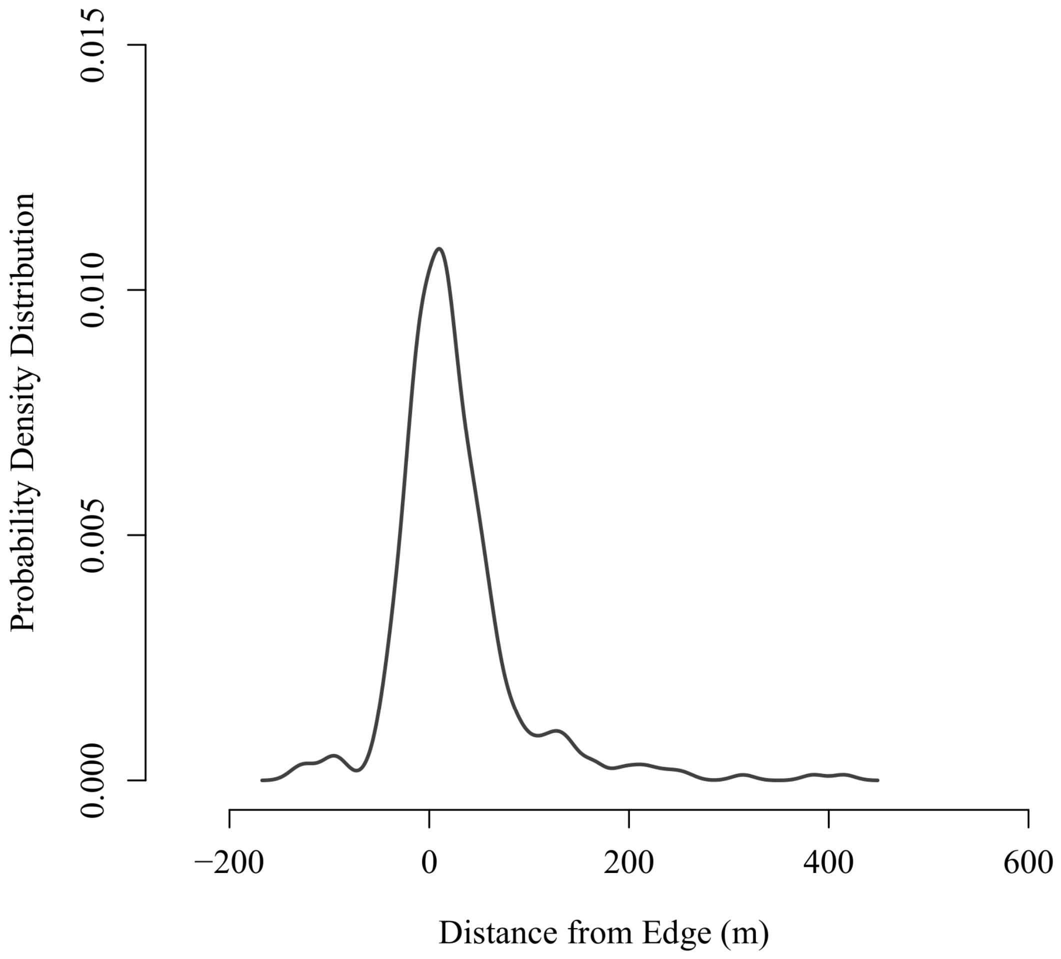

S2 Fig. Frequency of activity points as a function of distance to Forest edge. The graph shows the distribution of activity points versus the distance to forest edges, estimated via kernel density estimation. The grey line shows the probability density distribution of the collected points in the study area.
