# Supplementary material for: Effects of Land Cover on the Movement of Frugivorous Birds in a Heterogeneous Landscape
Source: PLoS One. 2016 Jun 3;11(6):e0156688. doi: 10.1371/journal.pone.0156688 (PMC4892584; doi:10.1371/journal.pone.0156688)
Supplement: S3 Fig — Influences of land use classes (a), sex (b) and species (c) on the average speeds of thrushes within fragmented landscapes of southeastern Brazil. (PDF) [file pone.0156688.s003.pdf]

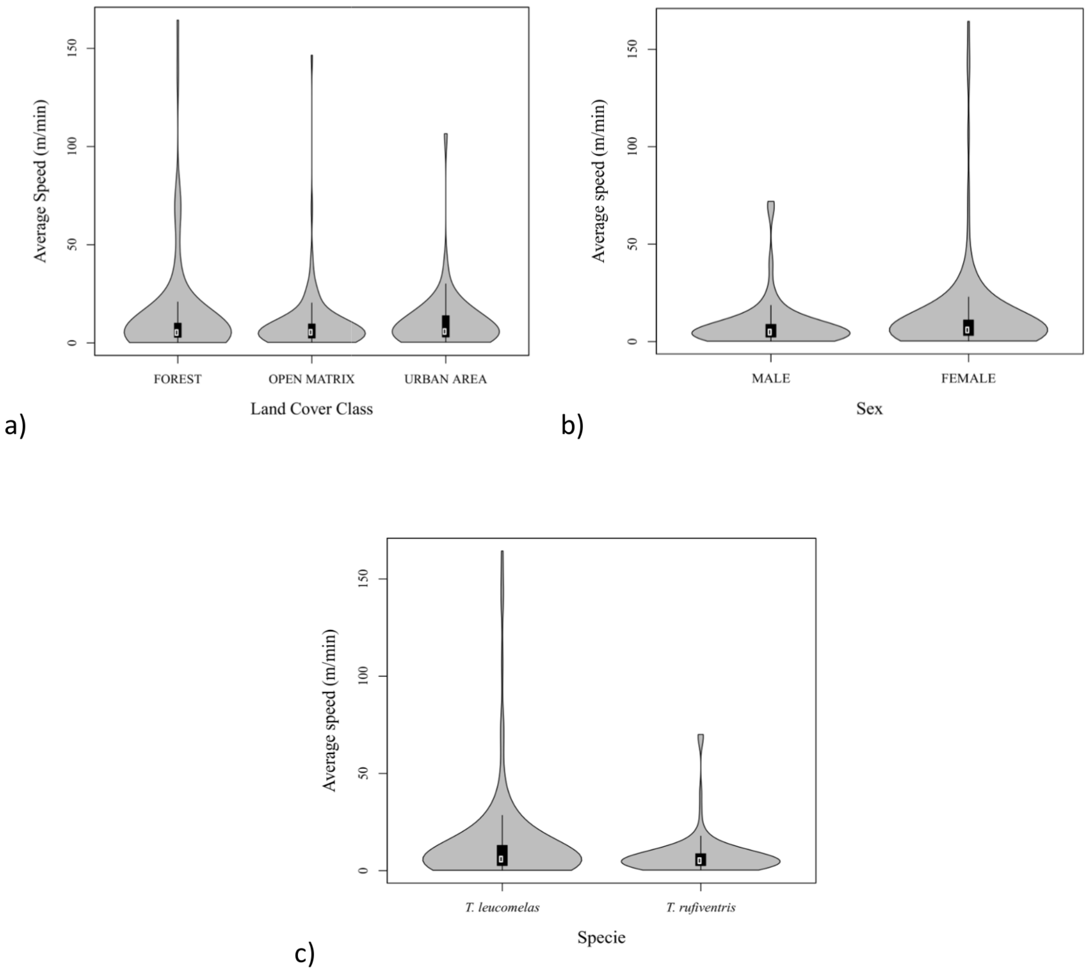

S3 fig. Influences of land use classes (a), sex (b) and species (c) on the average speeds of thrushes within fragmented landscapes of southeastern Brazil. a) Average speeds found in three cover classes (forests, urban areas and pasture/plantations), b) average speeds for males and females, c) average speeds for *T. rufiventris* and *T. leucomelas.*
