# Supplementary material for: Effects of Land Cover on the Movement of Frugivorous Birds in a Heterogeneous Landscape
Source: PLoS One. 2016 Jun 3;11(6):e0156688. doi: 10.1371/journal.pone.0156688 (PMC4892584; doi:10.1371/journal.pone.0156688)
Supplement: S1 File — (PDF) [file pone.0156688.s004.pdf]

S1 File. Full data from two species of thrushes of the genus Turdus sp. collected by using the radio telemetry methodology from June 2013 to June 2014 in the city limits of Itatiba, São Paulo, Brazil.

ID	Date/ hour	X estimate	Y estimate	Weight (g)	Sex*	Specie**
1	29/11/2013 07:16	320912	7459159	89	2	2
1	29/11/2013 07:47	321194	7459067	89	2	2
1	29/11/2013 08:02	321171	7459118	89	2	2
1	29/11/2013 08:17	321089	7459222	89	2	2
1	29/11/2013 08:35	321115	7459099	89	2	2
1	29/11/2013 08:41	321126	7459068	89	2	2
1	29/11/2013 08:53	321191	7459165	89	2	2
1	29/11/2013 09:10	321118	7459068	89	2	2
2	22/02/2014 07:01	320347	7460642	84	2	1
2	22/02/2014 07:15	320447	7460556	84	2	1
2	22/02/2014 07:30	320437	7460544	84	2	1
2	22/02/2014 08:00	320577	7460376	84	2	1
2	22/02/2014 08:15	320493	7460390	84	2	1
2	22/02/2014 08:30	320432	7460412	84	2	1
2	22/02/2014 08:45	320351	7460377	84	2	1
2	22/02/2014 09:00	320677	7460212	84	2	1
2	22/02/2014 09:15	320532	7460474	84	2	1
2	22/02/2014 09:45	320286	7460657	84	2	1
2	22/02/2014 10:25	320321	7460531	84	2	1
2	22/02/2014 13:30	320374	7460633	84	2	1
2	22/02/2014 13:45	320407	7460656	84	2	1
2	22/02/2014 14:00	320365	7460661	84	2	1
2	02/03/2014 07:00	320517	7460449	84	2	1
2	02/03/2014 07:10	320613	7460578	84	2	1
2	02/03/2014 07:45	320298	7460699	84	2	1
2	02/03/2014 07:55	320292	7460735	84	2	1
2	02/03/2014 08:15	320208	7460694	84	2	1
2	02/03/2014 08:25	320158	7460663	84	2	1
2	02/03/2014 08:35	320181	7460693	84	2	1
2	02/03/2014 08:45	320128	7460634	84	2	1
2	02/03/2014 08:55	320113	7460771	84	2	1
2	02/03/2014 09:05	320267	7460346	84	2	1
2	02/03/2014 09:30	320321	7460545	84	2	1
2	02/03/2014 09:50	320708	7460314	84	2	1
2	02/03/2014 10:00	320340	7460485	84	2	1
2	02/03/2014 10:20	320341	7460507	84	2	1
2	02/03/2014 10:30	320297	7460511	84	2	1
2	02/03/2014 10:40	320393	7460469	84	2	1
2	02/03/2014 12:00	320259	7460635	84	2	1
2	03/03/2014 08:00	320255	7460540	84	2	1
2	03/03/2014 08:20	320324	7460605	84	2	1
2	03/03/2014 08:30	320407	7460508	84	2	1

2	03/03/2014 08:40	320400	7460537	84	2	1
2	03/03/2014 08:50	320414	7460457	84	2	1
2	03/03/2014 09:10	320397	7460412	84	2	1
2	03/03/2014 09:20	320373	7460525	84	2	1
2	03/03/2014 09:30	320373	7460500	84	2	1
2	03/03/2014 09:50	320315	7460528	84	2	1
2	03/03/2014 10:30	320374	7460505	84	2	1
2	03/03/2014 10:50	320363	7460468	84	2	1
2	03/03/2014 11:00	320387	7460468	84	2	1
2	03/03/2014 11:10	320546	7460232	84	2	1
2	03/03/2014 11:20	320474	7460255	84	2	1
2	05/04/2014 07:02	320238	7460678	84	2	1
2	05/04/2014 07:45	320741	7461185	84	2	1
2	05/04/2014 08:05	320171	7460831	84	2	1
2	05/04/2014 08:15	320258	7460775	84	2	1
2	05/04/2014 09:20	320265	7460708	84	2	1
2	05/04/2014 09:30	320272	7460685	84	2	1
2	05/04/2014 09:54	320290	7460595	84	2	1
2	05/04/2014 10:04	320310	7460744	84	2	1
2	05/04/2014 10:14	320329	7460711	84	2	1
2	05/04/2014 10:54	320363	7460884	84	2	1
2	05/04/2014 13:00	320302	7460709	84	2	1
2	05/04/2014 13:10	320313	7460809	84	2	1
2	05/04/2014 13:20	320362	7460927	84	2	1
2	05/04/2014 13:30	320965	7460987	84	2	1
2	05/04/2014 13:40	320264	7460676	84	2	1
2	05/04/2014 14:00	320516	7460959	84	2	1
2	05/04/2014 14:10	320345	7460677	84	2	1
2	05/04/2014 14:20	321984	7460804	84	2	1
2	05/04/2014 14:30	320519	7460828	84	2	1
2	05/04/2014 14:40	320255	7460688	84	2	1
2	05/04/2014 15:08	320238	7460643	84	2	1
2	20/04/2014 16:10	320317	7460540	84	2	1
2	20/04/2014 16:20	320395	7460492	84	2	1
2	29/04/2014 06:58	320401	7460304	84	2	1
2	29/04/2014 07:43	320380	7460465	84	2	1
2	29/04/2014 07:58	320325	7460478	84	2	1
2	29/04/2014 08:13	320347	7460456	84	2	1
2	29/04/2014 08:28	320396	7460414	84	2	1
2	29/04/2014 08:43	320441	7460473	84	2	1
2	29/04/2014 08:58	320377	7460391	84	2	1
2	29/04/2014 09:13	320677	7460166	84	2	1
2	29/04/2014 09:28	320727	7460172	84	2	1
2	29/04/2014 09:58	320422	7460291	84	2	1
2	29/04/2014 10:13	320566	7460234	84	2	1
2	29/04/2014 10:43	320354	7460450	84	2	1
2	29/04/2014 13:41	320374	7460482	84	2	1

2	29/04/2014 13:57	320350	7460381	84	2	1
2	29/04/2014 14:12	320330	7460485	84	2	1
2	29/04/2014 14:27	320349	7460495	84	2	1
2	29/04/2014 14:42	320447	7460529	84	2	1
2	29/04/2014 14:57	320357	7460546	84	2	1
2	29/04/2014 15:12	320620	7460266	84	2	1
2	29/04/2014 15:27	320483	7460517	84	2	1
2	29/04/2014 15:42	320520	7460508	84	2	1
2	29/04/2014 15:57	320490	7460501	84	2	1
2	29/04/2014 16:12	320446	7460480	84	2	1
2	29/04/2014 16:27	320448	7460517	84	2	1
2	29/04/2014 16:42	320439	7460446	84	2	1
2	29/04/2014 16:57	320369	7460532	84	2	1
2	30/04/2014 07:03	320388	7460422	84	2	1
2	30/04/2014 07:18	320372	7460457	84	2	1
2	30/04/2014 07:33	320385	7460450	84	2	1
2	30/04/2014 08:18	320342	7460461	84	2	1
2	30/04/2014 08:28	320381	7460294	84	2	1
2	30/04/2014 10:03	320786	7460355	84	2	1
2	30/04/2014 14:55	320400	7460516	84	2	1
2	30/04/2014 15:25	320700	7460581	84	2	1
2	30/04/2014 15:55	320507	7460482	84	2	1
2	30/04/2014 16:10	320344	7460498	84	2	1
3	18/07/2013 08:00	321088	7459191	69	1	1
3	18/07/2013 09:00	321163	7459030	69	1	1
3	18/07/2013 09:15	321197	7459041	69	1	1
3	18/07/2013 09:30	321210	7459012	69	1	1
3	18/07/2013 10:45	321226	7459119	69	1	1
3	18/07/2013 13:55	321124	7459015	69	1	1
3	18/07/2013 14:40	321187	7459155	69	1	1
3	18/07/2013 14:55	321163	7459038	69	1	1
3	18/07/2013 15:10	321165	7459036	69	1	1
3	19/07/2013 09:00	321608	7458826	69	1	1
3	19/07/2013 09:30	322013	7458724	69	1	1
3	20/07/2013 08:45	322215	7457680	69	1	1
3	20/07/2013 09:45	321593	7459020	69	1	1
3	20/07/2013 10:30	321572	7458978	69	1	1
3	20/07/2013 14:05	321374	7458947	69	1	1
3	20/07/2013 14:35	321499	7458983	69	1	1
3	20/07/2013 14:50	321512	7458977	69	1	1
3	20/07/2013 15:05	321580	7458933	69	1	1
3	20/07/2013 16:05	321778	7458998	69	1	1
3	20/07/2013 16:20	321837	7459005	69	1	1
3	20/07/2013 16:35	321748	7458970	69	1	1
3	08/08/2013 15:01	321394	7459065	69	1	1
3	09/08/2013 07:44	321532	7458942	69	1	1
3	09/08/2013 08:23	321552	7458985	69	1	1

3	09/08/2013 10:08	321392	7459322	69	1	1
3	09/08/2013 10:19	321388	7459315	69	1	1
3	10/08/2013 13:00	321437	7459236	69	1	1
3	08/09/2013 16:32	321063	7459585	69	1	1
3	08/09/2013 16:47	321178	7458981	69	1	1
3	08/09/2013 17:02	321180	7458969	69	1	1
3	08/09/2013 17:17	321160	7458970	69	1	1
3	08/09/2013 17:32	321142	7459000	69	1	1
3	09/09/2013 07:10	321153	7458968	69	1	1
3	09/09/2013 08:30	321777	7459002	69	1	1
3	09/09/2013 09:35	321831	7459399	69	1	1
4	05/04/2014 07:28	320276	7460570	98	2	2
4	05/04/2014 07:58	320266	7460632	98	2	2
4	05/04/2014 08:08	320281	7460679	98	2	2
4	05/04/2014 09:23	320202	7460714	98	2	2
4	05/04/2014 09:33	320249	7460660	98	2	2
4	05/04/2014 09:43	320312	7460726	98	2	2
4	05/04/2014 10:25	320348	7460694	98	2	2
4	05/04/2014 10:35	320319	7460733	98	2	2
4	05/04/2014 10:45	320294	7460730	98	2	2
4	05/04/2014 10:55	320288	7460692	98	2	2
4	05/04/2014 12:53	320212	7460604	98	2	2
4	05/04/2014 13:03	320235	7460554	98	2	2
4	05/04/2014 13:13	320188	7460454	98	2	2
4	05/04/2014 14:23	320312	7460541	98	2	2
4	05/04/2014 14:33	320357	7460577	98	2	2
4	05/04/2014 15:50	320388	7460548	98	2	2
4	20/04/2014 14:10	320319	7460491	98	2	2
4	20/04/2014 14:22	320278	7460579	98	2	2
4	20/04/2014 14:42	320312	7460539	98	2	2
4	20/04/2014 15:02	320345	7460546	98	2	2
4	20/04/2014 15:12	320366	7460581	98	2	2
4	20/04/2014 15:22	320438	7460667	98	2	2
4	20/04/2014 15:32	320379	7460574	98	2	2
4	20/04/2014 15:42	320335	7460563	98	2	2
4	20/04/2014 16:12	320429	7460559	98	2	2
4	29/04/2014 07:18	320319	7460512	98	2	2
4	20/06/2014 14:52	320478	7460730	98	2	2
4	21/06/2014 07:30	320314	7460687	98	2	2
4	21/06/2014 07:45	320270	7460685	98	2	2
4	21/06/2014 08:40	320500	7460727	98	2	2
4	21/06/2014 09:10	320366	7460773	98	2	2
4	21/06/2014 09:40	320647	7460671	98	2	2
5	05/04/2014 07:10	320307	7460632	83	2	1
5	05/04/2014 07:20	320336	7460590	83	2	1
5	05/04/2014 07:30	320435	7460651	83	2	1
5	05/04/2014 07:40	321439	7461587	83	2	1

5	05/04/2014 08:30	320309	7460673	83	2	1
5	05/04/2014 09:25	320307	7460693	83	2	1
5	05/04/2014 09:35	320254	7460615	83	2	1
5	05/04/2014 09:57	320631	7460467	83	2	1
5	05/04/2014 10:07	320375	7460492	83	2	1
5	05/04/2014 10:17	320605	7460380	83	2	1
5	05/04/2014 10:27	320354	7460371	83	2	1
5	05/04/2014 13:26	320298	7460590	83	2	1
5	05/04/2014 13:36	320292	7460589	83	2	1
5	05/04/2014 13:56	320517	7460718	83	2	1
5	05/04/2014 14:06	320329	7460597	83	2	1
5	05/04/2014 14:16	320582	7460480	83	2	1
5	05/04/2014 14:26	320398	7460533	83	2	1
5	05/04/2014 14:46	320330	7460612	83	2	1
5	05/04/2014 14:13	320280	7460585	83	2	1
5	05/04/2014 15:23	320315	7460559	83	2	1
5	05/04/2014 15:33	320259	7460589	83	2	1
5	05/04/2014 15:53	320311	7460646	83	2	1
5	05/04/2014 16:03	320316	7460609	83	2	1
5	29/04/2014 07:08	320201	7460649	83	2	1
5	29/04/2014 14:17	320233	7460312	83	2	1
5	29/04/2014 15:17	320282	7460514	83	2	1
5	29/04/2014 16:02	320322	7460449	83	2	1
5	29/04/2014 17:02	320305	7460448	83	2	1
5	30/04/2014 07:27	320264	7460300	83	2	1
6	08/08/2013 13:36	319370	7458715	78	1	2
6	08/08/2013 13:51	319355	7458657	78	1	2
6	08/08/2013 14:48	319390	7458659	78	1	2
6	08/08/2013 15:03	319396	7458749	78	1	2
6	08/08/2013 15:31	319117	7458814	78	1	2
6	08/08/2013 15:46	319353	7458916	78	1	2
6	08/08/2013 16:01	319366	7458909	78	1	2
6	08/08/2013 16:16	319361	7458913	78	1	2
6	10/08/2013 07:43	319513	7458843	78	1	2
6	10/08/2013 07:58	319465	7458716	78	1	2
6	10/08/2013 08:13	319396	7458745	78	1	2
6	10/08/2013 09:50	319449	7458682	78	1	2
6	24/08/2013 06:47	319423	7458719	78	1	2
6	24/08/2013 07:02	319435	7458715	78	1	2
6	24/08/2013 07:17	319428	7458686	78	1	2
6	24/08/2013 07:35	319433	7458718	78	1	2
6	24/08/2013 07:50	319421	7458734	78	1	2
6	24/08/2013 08:05	319397	7458775	78	1	2
6	24/08/2013 08:20	319392	7458771	78	1	2
6	24/08/2013 08:35	319412	7458760	78	1	2
6	24/08/2013 09:35	319256	7458758	78	1	2
6	24/08/2013 09:50	319380	7458721	78	1	2

6	24/08/2013 10:05	319325	7458737	78	1	2
6	25/08/2013 06:55	318410	7458922	78	1	2
6	25/08/2013 07:10	319416	7458701	78	1	2
6	07/09/2013 15:40	318838	7459110	78	1	2
6	07/09/2013 15:55	319247	7458662	78	1	2
6	07/09/2013 16:05	319331	7458641	78	1	2
6	08/09/2013 08:26	319139	7458760	78	1	2
6	08/09/2013 08:41	319352	7458641	78	1	2
6	08/09/2013 08:56	319330	7458661	78	1	2
6	08/09/2013 10:11	319121	7458681	78	1	2
6	08/09/2013 10:26	319362	7458640	78	1	2
6	20/09/2013 10:19	319245	7458590	78	1	2
6	14/12/2013 14:00	319192	7458510	78	1	2
6	14/12/2013 14:15	319237	7458498	78	1	2
6	15/12/2013 15:32	319199	7458726	78	1	2
7	24/06/2013 09:51	321195	7459142	73	1	2
7	06/07/2013 07:52	321328	7459027	73	1	2
7	06/07/2013 08:27	321336	7459033	73	1	2
7	06/07/2013 08:45	321472	7459009	73	1	2
7	06/07/2013 12:38	321388	7459062	73	1	2
7	06/07/2013 13:00	320892	7459123	73	1	2
7	06/07/2013 13:17	321114	7458999	73	1	2
7	06/07/2013 13:31	321178	7459053	73	1	2
7	06/07/2013 14:27	321445	7459050	73	1	2
7	06/07/2013 14:50	321338	7459084	73	1	2
7	06/07/2013 15:05	321379	7459126	73	1	2
7	07/07/2013 08:25	321026	7459042	73	1	2
7	07/07/2013 08:40	321047	7459042	73	1	2
7	07/07/2013 08:59	320989	7458947	73	1	2
7	07/07/2013 09:15	321079	7458914	73	1	2
7	07/07/2013 09:55	321475	7458951	73	1	2
7	07/07/2013 10:25	321194	7458964	73	1	2
7	07/07/2013 13:52	321227	7458935	73	1	2
7	07/07/2013 14:05	321327	7458905	73	1	2
7	07/07/2013 14:15	321417	7458951	73	1	2
7	07/07/2013 14:27	321470	7459032	73	1	2
7	07/07/2013 14:39	321543	7458977	73	1	2
7	07/07/2013 14:49	321516	7458978	73	1	2
7	07/07/2013 15:03	321520	7458966	73	1	2
7	07/07/2013 15:15	321583	7458960	73	1	2
7	07/07/2013 15:40	321456	7459019	73	1	2
7	09/07/2013 07:27	321121	7459066	73	1	2
7	09/07/2013 07:42	321190	7459010	73	1	2
7	09/07/2013 07:58	321095	7458966	73	1	2
7	09/07/2013 08:28	321090	7459033	73	1	2
7	09/07/2013 08:43	321209	7459087	73	1	2
7	09/07/2013 08:58	321153	7459055	73	1	2

7	09/07/2013 09:13	321171	7459030	73	1	2
7	09/07/2013 09:28	321158	7459065	73	1	2
7	09/07/2013 09:43	321144	7459011	73	1	2
7	09/07/2013 09:58	321158	7459093	73	1	2
7	09/07/2013 12:38	321250	7459096	73	1	2
7	09/07/2013 12:53	321170	7459092	73	1	2
7	09/07/2013 13:08	321123	7459080	73	1	2
7	09/07/2013 13:38	321233	7459075	73	1	2
7	09/07/2013 14:08	321131	7459035	73	1	2
7	09/07/2013 14:23	321147	7459075	73	1	2
7	09/07/2013 14:38	321148	7459053	73	1	2
7	09/07/2013 15:08	321091	7459036	73	1	2
8	25/08/2013 07:29	319652	7458690	78	1	2
8	25/08/2013 07:07	319553	7458631	78	1	2
8	25/08/2013 06:52	319491	7458726	78	1	2
8	23/08/2013 09:23	319511	7458655	78	1	2
8	23/08/2013 08:42	319796	7458451	78	1	2
8	23/08/2013 08:12	319595	7458653	78	1	2
8	23/08/2013 07:57	319547	7458672	78	1	2
8	23/08/2013 07:12	319527	7458670	78	1	2
8	23/08/2013 06:57	319507	7458686	78	1	2
8	23/08/2013 06:42	319543	7458658	78	1	2
8	23/08/2013 16:29	319355	7458697	78	1	2
8	23/08/2013 16:14	319230	7458702	78	1	2
8	23/08/2013 15:59	319388	7458680	78	1	2
8	23/08/2013 15:44	318995	7458917	78	1	2
8	23/08/2013 15:14	319245	7458742	78	1	2
8	23/08/2013 14:57	318977	7458855	78	1	2
8	23/08/2013 14:42	319095	7458787	78	1	2
8	23/08/2013 14:27	319337	7458727	78	1	2
8	23/08/2013 14:12	319254	7458764	78	1	2
9	20/09/2013 06:46	319394	7458748	94	1	1
9	20/09/2013 07:15	319083	7458827	94	1	1
9	20/09/2013 07:35	319281	7458628	94	1	1
9	20/09/2013 07:50	318265	7458991	94	1	1
9	20/09/2013 08:38	319273	7458699	94	1	1
9	20/09/2013 08:56	319282	7458626	94	1	1
9	20/09/2013 09:16	319237	7458878	94	1	1
9	20/09/2013 09:30	319228	7458867	94	1	1
9	20/09/2013 09:45	319231	7458557	94	1	1
9	15/11/2013 08:13	319105	7458641	94	1	1
9	15/11/2013 08:28	319068	7458645	94	1	1
9	15/11/2013 09:58	319088	7458611	94	1	1
9	15/11/2013 10:13	319120	7458625	94	1	1
10	22/02/2014 07:28	321158	7460134	70	1	2
10	22/02/2014 10:23	320360	7460528	70	1	2
10	22/02/2014 10:40	320458	7460549	70	1	2

10	22/02/2014 11:10	320244	7460627	70	1	2
10	22/02/2014 13:10	320404	7460700	70	1	2
10	22/02/2014 13:25	321448	7460820	70	1	2
10	22/02/2014 13:40	320437	7460704	70	1	2
10	22/02/2014 13:55	320482	7460721	70	1	2
10	22/02/2014 14:10	320428	7460707	70	1	2
10	02/03/2014 07:10	320430	7460642	70	1	2
10	02/03/2014 07:20	320517	7460547	70	1	2

The thrushes collected were tagged with radio transmitters, and a blood sample was collected for sex identification. The data are in projection UTM (Universal Transverse Mercator), WSG 1984 UTM Zone 23S.

* male = 1 and female = 2

** *Turdus leucomelas* = 1 and *Turdus rufiventris* = 2
