# Supplementary material for: Effects of Land Cover on the Movement of Frugivorous Birds in a Heterogeneous Landscape
Source: PLoS One. 2016 Jun 3;11(6):e0156688. doi: 10.1371/journal.pone.0156688 (PMC4892584; doi:10.1371/journal.pone.0156688)
Supplement: S1 Table — (PDF) [file pone.0156688.s005.pdf]

S1 Table. Individuals of *Turdus leucomelas* and *Turdus rufiventris* followed by radio telemetry from June 2013 to June 2014 in the city limits of Itatiba, São Paulo.

**Individuals**	**Weigh (g)**	**Species**	**Sex**	**Sample period**	**Sample size (Average speeds)**	**Sample size (Turning angles)**
1	*89*	*Turdus rufiventris*	female	29/11/2013	7	6
2	*84*	*Turdus leucomelas*	female	22/02/2014 to 30/04/2014	93	86
3	*69*	*Turdus leucomelas*	male	18/07/2013 to 9/9/2013	25	19
4	*98*	*Turdus rufiventris*	female	05/05/2014 to 30/04/2014	27	24
5	*83*	*Turdus leucomelas*	female	05/04/2014 to 30/04/2014	24	23
6	*76*	*Turdus leucomelas*	male	8/8/2013 to 15/12/2013	28	21
7	*73*	*Turdus rufiventris*	male	24/06/2103 to 9/7/2013	37	34
8	*78*	*Turdus rufiventris*	male	23/8/2013 to 25/8/2013	11	9
9	*94*	*Turdus leucomelas*	male	20/9/2013 to 15/11/2013	16	14
10	*70*	*Turdus rufiventris*	male	22/02/2014 to 02/03/2014	7	6
					275	242

The thrushes collected were tagged with radio transmitters, and a blood sample was collected for sex identification. The tracking period varied between individuals captured due to the limitations of radio telemetry in mountainous environments.
