# Supplementary material for: Effects of Land Cover on the Movement of Frugivorous Birds in a Heterogeneous Landscape
Source: PLoS One. 2016 Jun 3;11(6):e0156688. doi: 10.1371/journal.pone.0156688 (PMC4892584; doi:10.1371/journal.pone.0156688)
Supplement: S1 Text — (PDF) [file pone.0156688.s006.pdf]

S1 Text. Movement analysis script for thrushes in fragmented landscapes.

```
##########################################################
#
# Movement analysis for thrushes in
# fragmented landscapes
#
# Natalia Stefanini Silveira - nat.stefanini at gmail.com
# Bernardo Niebuhr - bernardo_brandaum at yahoo.com.br
# Jul. 2014
#
# Reference: Silveira et al. 2016.
# Effects of land cover on the movement of frugivorous
# birds in a heterogeneous  landscape.
#
# No rights reserved - feel free to modify and share
##########################################################

if(!require(adehabitatLT)) install.packages("adehabitatLT", dep=T); library(adehabitatLT)
if(!require(bbmle)) install.packages("bbmle", dep=T); library(bbmle)
if(!require(circular)) install.packages("circular", dep=T); library(circular)
if(!require(R2jags)) install.packages("R2jags", dep=T); library(R2jags)

###################
# Loading and organizing data
rm(list=ls())
```

```
# Changing working directory
#setwd("")

# Reading data
data <- read.table("dados_final.csv", sep=",",dec=".", header=TRUE)

head(data)
str(data)
names(data)=c("nome", "sexo", "data","hora", "especie", "xestimate", "yestimate",
        "id_poligono", "classe", "classe_bin", "peso", "sitio", "dist_borda")

data$prox_borda <- ifelse(data$dist_borda > 0, data$dist_borda, -data$dist_borda)

# Transforming data into ltraj class
data$data<-as.character(data$data)
data$hora<-as.character(data$hora)

da <- paste(data$data, data$hora)
da

bursts <- paste(data$nome, data$data, sep="_")

da1 <- as.POSIXct(strptime(da, format="%Y-%m-%d %H:%M"))

path <- as.ltraj(xy = data[,c("xestimate", "yestimate")], date = da1, id = data$nome,
         burst=bursts, typeII=T, infolocs=data[,c("sexo", "especie", "id_poligono",
                                 "classe", "classe_bin",
```

```
                                        "peso", "sitio", "dist_borda",
                                        "prox_borda")])

# 3 classes
path.df$class3 <- as.character(path.df$classe_bin)
path.df$class3[path.df$classe == "res"] <- "URB"
path.df$class3 <- as.factor(path.df$class3)

# Deleting fixes with dt > 2h = 7200s
path.df <- path.df[!is.na(path.df$dist),]
path.df <- path.df[path.df$dt < 7200,]

# Separating dependent variables
dist <- path.df$dist
time <- path.df$dt[!is.na(path.df$dt)]

# Average speed
velo <- dist/time*60
# Turning angles
angle <- path.df$rel.angle[!is.na(path.df$rel.angle)]

# Separating independent variables
# for Y = Mean speed
class.bin.v = path.df$classe_bin
species.v = as.factor(path.df$especie)
site.v = as.factor(path.df$sitio)
sex.v = as.factor(path.df$sexo)
```

```
class3.v = path.df$class3

distedge.v = path.df$dist_borda

proxedge.v = path.df$prox_borda

id <- path.df$id


##############

# Species

# 1 = T. leucomelas

# 2 = T. rufiventris


##############

# Sex

# 1 = male

# 2 = female


##############

# Class

# 1 = forest

# 2 = matrix


##### MODELS ######


# Y = mean speed or turning angles


####################

# For Y = Mean speed
```

```
####################
# Exponential models
####################

### Defining models
LLexp <- function(lambda){
  -sum(dexp(velo, rate=lambda, log=T))
}

# Univariate models
LLexp1 <- function(a, b){
  lambda = c(a, b)[class.bin.v]
  -sum(dexp(velo, rate=lambda, log=T))
}

LLexp2 <- function(a, b, c){
  lambda = c(a, b, c)[class3.v]
  -sum(dexp(velo, rate=lambda, log=T))
}

LLexp3 <- function(a, b){
  lambda = c(a, b)[species.v]
  -sum(dexp(velo, rate=lambda, log=T))
}

LLexp4 <- function(a, b){
  lambda = c(a, b)[sex.v]
```

```
  -sum(dexp(velo, rate=lambda, log=T))
}

LLexp5 <- function(a, b){
  lambda = exp(a + b*proxedge.v)
  -sum(dexp(velo, rate=lambda, log=T))
}

# Multivariate models
LLexp6 <- function(a, b, c){
  lambda = c(a, b)[sex.v] + c(0, c)[class.bin.v]
  -sum(dexp(velo, rate=lambda, log=T))
}

LLexp7 <- function(a, b, c, d){
  lambda = c(a, b)[sex.v] + c(0, c, d)[class3.v]
  -sum(dexp(velo, rate=lambda, log=T))
}

LLexp8 <- function(a, b, c){
  lambda = c(a, b)[species.v] + c(0, c)[class.bin.v]
  -sum(dexp(velo, rate=lambda, log=T))
}

LLexp9 <- function (a, b, c, d){
  lambda = c(a, b)[species.v] + c(0, c, d)[class3.v]
  -sum(dexp(velo, rate=lambda, log=T))
```

```
}

LLexp10 <- function(a, b, c, d) {
  lambda = exp(c(a, b)[class.bin.v] + c(c, d)[class.bin.v]*proxedge.v)
  -sum(dexp(velo, rate=lambda, log=T))
}

LLexp11 <- function(a, b, c, d, e, f) {
  lambda = exp(c(a, b, c)[class3.v] + c(d, e, f)[class3.v]*proxedge.v)
  -sum(dexp(velo, rate=lambda, log=T))
}

### Fitting models
mexp <- mle2(LLexp, start=list(lambda=1/mean(velo)))

mexp1 <- mle2(LLexp1, start=list(a = 1/mean(velo), b = 1/mean(velo)))

mexp2 <- mle2(LLexp2, start=list(a = 1/mean(velo), b = 1/mean(velo), c = 
1/mean(velo)))

mexp3 <- mle2(LLexp3, start=list(a = 1/mean(velo), b = 1/mean(velo)))

mexp4 <- mle2(LLexp4, start=list(a = 1/mean(velo), b = 1/mean(velo)))

a.tent = log(coef(glm(velo ~ proxedge.v, family = "Gamma"))[1])
b.tent = coef(glm(velo ~ proxedge.v, family = "Gamma"))[2]
mexp5 <- mle2(LLexp5, start=list(a = a.tent, b = b.tent))
```

```
mexp6 <- mle2(LLexp6, start=list(a = 1/mean(velo), b = 1/mean(velo), c =
1/mean(velo)))

mexp7 <- mle2(LLexp7, start=list(a = 1/mean(velo), b = 1/mean(velo), c =
1/mean(velo), d = 1/mean(velo)))

mexp8 <- mle2(LLexp8, start=list(a = 1/mean(velo), b = 1/mean(velo), c =
1/mean(velo)))

mexp9 <- mle2(LLexp9, start=list(a = 1/mean(velo), b = 1/mean(velo), c =
1/mean(velo), d = 1/mean(velo)))

a.tent = log(coef(glm(velo ~ proxedge.v, family = "Gamma"))[1])

b.tent = coef(glm(velo ~ proxedge.v, family = "Gamma"))[2]

mexp10 <- mle2(LLexp10, start=list(a = a.tent, b = a.tent, c = b.tent, d = b.tent))

a.tent = log(coef(glm(velo ~ proxedge.v, family = "Gamma"))[1])

b.tent = coef(glm(velo ~ proxedge.v, family = "Gamma"))[2]

mexp11 <- mle2(LLexp11, start=list(a = a.tent, b = a.tent, c = a.tent, d = b.tent, e =
b.tent, f = b.tent))

# coeff <- coef(mexp11)

# matmax <- max(proxedge.v[class.bin.v != "MATA"])

# habmax <- max(proxedge.v[class.bin.v == "MATA"])

#

# # all classes together, different intercepts

# plot(0, 0, type = "n", xlim = c(-matmax, habmax), ylim = c(0, max(velo)), xlab =
"DistÃ¢ncia da borda",
```

```
#      ylab = "Velocidade mÃ©dia (m/min)")
# abline(v = 0, col = "dark green", lty = 2)
# curve(1/(exp(coeff[1] + coeff[4]*x)), 0, habmax, col = "dark green", add=T)
# curve(1/(exp(coeff[3] - coeff[6]*x)), -matmax, 0, col = "red", add = T)
# curve(1/(exp(coeff[2] - coeff[5]*x)), -matmax, 0, col = "gold", add = T)

##############################
# Weibull distribution models
##############################

### Defining models

LLwei <- function(forma, escala) {
  -sum(dweibull(velo, shape=forma, scale=escala, log=T))
}

# Univariate models
LLwei1 <- function(a, b, escala) {
   forma = exp(c(a, b)[class.bin.v])
   -sum(dweibull(velo, shape=forma, scale=escala, log=T))
}

LLwei2 <- function(a, b, c, escala) {
  forma = exp(c(a, b, c)[class3.v])
  -sum(dweibull(velo, shape=forma, scale=escala, log=T))
}
```

```
}

LLwei3 <- function(a, b, escala) {
  forma = exp(c(a, b)[species.v])
  -sum(dweibull(velo, shape=forma, scale=escala, log=T))
}

LLwei4 <- function(a, b, escala) {
  forma = exp(c(a, b)[sex.v])
  -sum(dweibull(velo, shape=forma, scale=escala, log=T))
}

LLwei5 <- function(a, b, escala) {
  forma = exp(a + b*proxedge.v)
  -sum(dweibull(velo, shape=forma, scale=escala, log=T))
}

# Multivariate models
LLwei6 <- function(a, b, c, escala) {
  forma = exp(c(a, b)[sex.v] + c(0, c)[class.bin.v])
  -sum(dweibull(velo, shape=forma, scale=escala, log=T))
}

LLwei7 <- function(a, b, c, d, escala) {
  forma = exp(c(a, b)[sex.v] + c(0, c, d)[class3.v])
  -sum(dweibull(velo, shape=forma, scale=escala, log=T))
}

LLwei8 <- function(a, b, c, escala) {
```

```
  forma = exp(c(a, b)[species.v] + c(0, c)[class.bin.v])
  -sum(dweibull(velo, shape=forma, scale=escala, log=T))
}

LLwei9 <- function(a, b, c, d, escala) {
  forma = exp(c(a, b)[species.v] + c(0, c, d)[class3.v])
  -sum(dweibull(velo, shape=forma, scale=escala, log=T))
}

LLwei10 <- function(a, b, c, d, escala) {
  forma = exp(c(a, b)[class.bin.v] + c(c, d)[class.bin.v]*proxedge.v)
  -sum(dweibull(velo, shape=forma, scale=escala, log=T))
}

LLwei11 <- function(a, b, c, d, e, f, escala) {
  forma = exp(c(a, b, c)[class3.v] + c(d, e, f)[class3.v]*proxedge.v)
  -sum(dweibull(velo, shape=forma, scale=escala, log=T))
}

### Fitting models to data
mwei <- mle2(LLwei, start=list(forma = mean(velo)^2/var(velo), escala =
var(velo)/mean(velo)))
esc <- coef(mwei)[2]

mwei1 <- mle2(LLwei1, start=list(a = 1, b = 1, escala = esc))

mwei2 <- mle2(LLwei2, start=list(a = 1, b = 1, c = 1, escala = esc))
```

```
mwei3 <- mle2(LLwei3, start=list(a = 1, b = 1, escala = esc))

mwei4 <- mle2(LLwei4, start=list(a = 1, b = 1, escala = esc))

mwei5 <- mle2(LLwei5, start=list(a = 0, b = 0, escala = esc))

mwei6 <- mle2(LLwei6, start=list(a = 0, b = 0, c = 0, escala = esc))

mwei7 <- mle2(LLwei7, start=list(a = 0, b = 0, c = 0, d = 0, escala = esc))

mwei8 <- mle2(LLwei8, start=list(a = 0, b = 0, c = 0, escala = esc))

mwei9 <- mle2(LLwei9, start=list(a = 0, b = 0, c = 0, d = 0, escala = esc))

mwei10 <- mle2(LLwei10, start=list(a = 0, b = 0, c = 0, d = 0, escala = esc))

mwei11 <- mle2(LLwei11, start=list(a = 0, b = 0, c = 0, d = 0, e = 0, f = 0, escala = 
esc))

##########################
# Levy distribution models
##########################

# Power-law distribution
dpowlaw <- function(x, alfa, xmin, log=FALSE){
  c <- (alfa-1)*xmin^(alfa-1)
```

```
  if(log) ifelse(x < xmin, 0, log(c*x^(-alfa)))
  else ifelse(x < xmin, 0, c*x^(-alfa))
}

# integrate(dpowlaw, -Inf, Inf, alfa=2, xmin=1)
# curve(dpowlaw(x, alfa=2.5, xmin=10), from=0, to=100, log="")
# curve(dpowlaw(x, alfa=2.5, xmin=1), from=1, to=100, log="xy")

### Defining models

LLlevy <- function(mu, xmin){
  -sum(dpowlaw(velo, alfa=mu, xmin=xmin, log=T))
}

### Fitting models to data
mlevy <- mle2(LLlevy, start=list(mu=2), fixed=list(xmin=min(velo)),
              method = "L-BFGS-B", lower=c(mu = 1.1), upper=c(mu = 4))

######################
#Truncated Levy models
######################

# Truncated power law function
dtpowlaw <- function(x, alfa, xmin, xmax, log=FALSE){
  c <- (alfa-1)*xmin^(alfa-1)/(1 - (xmin/xmax)^(alfa-1))
  if(log) ifelse(x < xmin | x > xmax, 0, log(c*x^(-alfa)))
  else ifelse(x < xmin | x > xmax, 0, c*x^(-alfa))
```

```
}


# integrate(dtpowlaw, -Inf, Inf, alfa=0.5, xmin=1, xmax=100)

# curve(dtpowlaw(x, alfa=1.5, xmin=1, xmax = 15), from=0, to=16, log="")


### Defining models


LLtlevy <- function(mu, xmin, xmax){

  -sum(dtpowlaw(velo, alfa=mu, xmin=xmin, xmax=xmax, log=T))

}


### Fitting models to data

mtlevy <- mle2(LLtlevy, start=list(mu=2), fixed=list(xmin=min(velo),
xmax=max(velo)+10),

              method = "L-BFGS-B", lower=c(mu = 1.0001), upper=c(mu = 5))



### Comparing all models

(comp <- AICctab(mexp, mexp1, mexp2, mexp3, mexp4, mexp5, mexp6, mexp7,

                mexp8, mexp9, mexp10, mexp11,

                mwei, mwei1, mwei2, mwei3, mwei4, mwei5, mwei6, mwei7,

                mwei8, mwei9, mwei10, mwei11,

                mlevy, mtlevy,

                base=T, weights=T, nobs=length(velo)))



########################
```

```
# For Y = Turning angles


#############################
# Wrapped Cauchy distribution
#############################


# Defining the function with log
dwcauchy <- function(theta, mu, rho, log=FALSE) {
  if(log) log(dwrpcauchy(theta, mu=mu, rho=rho))
  else dwrpcauchy(theta, mu=mu, rho=rho)
}
# curve(dwcauchy(x, 0, 0.5), -pi, pi)
# curve(dwcauchy(x, 0, 0.00001), -pi, pi, add=T)
# curve(dwcauchy(x, pi, 0.2), -pi, pi, add=T)
# integrate(dwcauchy, lower = -pi, upper = pi, mu = 0, rho = 0.5)


### Defining models


LLcauchy <- function(mu, rho) {
  -sum(dwcauchy(angle, mu, rho, log=T))
}


# Univariate models
LLcauchy1 <- function(a, b, rho) {
   mu =  c(a, b)[class.bin.a]
   -sum(dwcauchy(angle, mu, rho=rho, log=T))
}
```

```
}

LLcauchy2 <- function(a, b, c, rho) {
  mu =  c(a, b, c)[class3.a]
  -sum(dwcauchy(angle, mu, rho=rho, log=T))
}

LLcauchy3 <- function(a, b, rho) {
  mu = c(a, b)[species.a]
  -sum(dwcauchy(angle, mu, rho=rho, log=T))
}

LLcauchy4 <- function(a, b, rho) {
  mu = c(a, b)[sex.a]
  -sum(dwcauchy(angle, mu, rho=rho, log=T))
}

LLcauchy5 <- function(a, b, rho) {
  mu = a + b*proxedge.a
  -sum(dwcauchy(angle, mu, rho=rho, log=T))
}

# Multivariate models
LLcauchy6 <- function(a, b, c, rho) {
  mu = c(a, b)[sex.a] + c(0, c)[class.bin.a]
  -sum(dwcauchy(angle, mu, rho=rho, log=T))
}
```

```
LLcauchy7 <- function(a, b, c, d, rho) {
  mu = c(a, b)[sex.a] + c(0, c, d)[class3.a]
  -sum(dwcauchy(angle, mu, rho=rho, log=T))
}

LLcauchy8 <- function(a, b, c, rho) {
  mu = c(a, b)[species.a] + c(0, c)[class.bin.a]
  -sum(dwcauchy(angle, mu, rho=rho, log=T))
}

LLcauchy9 <- function(a, b, c, d, rho) {
  mu = c(a, b)[species.a] + c(0, c, d)[class3.a]
  -sum(dwcauchy(angle, mu, rho=rho, log=T))
}

LLcauchy10 <- function(a, b, c, d, rho) {
  mu =  c(a, b)[class.bin.a] + c(c, d)[class.bin.a]*proxedge.a
  -sum(dwcauchy(angle, mu, rho=rho, log=T))
}

LLcauchy11 <- function(a, b, c, d, e, f, rho) {
  mu =  c(a, b, c)[class3.a] + c(d, e, f)[class3.a]*proxedge.a
  -sum(dwcauchy(angle, mu, rho=rho, log=T))
}

### Fitting models to data
mcauchy <- mle2(LLcauchy, start=list(mu = pi, rho = 0.5),
```

```
        method = "L-BFGS-B", lower=c(mu = -2*pi, rho = 0),
        upper=c(mu = 2*pi, rho = 1))

mcauchy1 <- mle2(LLcauchy1, start=list(a = pi, b = pi, rho = 0.5),
         method = "L-BFGS-B", lower=c(a = -2*pi, b = -2*pi, rho = 0),
         upper=c(a = 2*pi, b = 2*pi, rho = 1))

mcauchy2 <- mle2(LLcauchy2, start=list(a = pi, b = pi, c = pi, rho = 0.5),
          method = "L-BFGS-B", lower=c(a = -2*pi, b = -2*pi, c = -2*pi, rho = 0),
          upper=c(a = 2*pi, b = 2*pi, c = 2*pi, rho = 1))

mcauchy3 <- mle2(LLcauchy3, start=list(a = pi, b = pi, rho = 0.5),
         method = "L-BFGS-B", lower=c(a = -2*pi, b = -2*pi, rho = 0),
         upper=c(a = 2*pi, b = 2*pi, rho = 1))

mcauchy4 <- mle2(LLcauchy4, start=list(a = pi, b = pi, rho = 0.5),
         method = "L-BFGS-B", lower=c(a = -2*pi, b = -2*pi, rho = 0.01),
         upper=c(a = 2*pi, b = 2*pi, rho = 0.99))

mcauchy5 <- mle2(LLcauchy5, start=list(a = pi, b = 0, rho = 0.5),
          method = "L-BFGS-B", lower=c(a = -2*pi, b = -0.1, rho = 0.01),
          upper=c(a = 2*pi, b = 0.1, rho = 0.99))

mcauchy6 <- mle2(LLcauchy6, start=list(a = pi, b = pi, c = 0, rho = 0.5),
         method = "L-BFGS-B", lower=c(a = -2*pi, b = -2*pi, c = -pi, rho = 0.01),
         upper=c(a = 2*pi, b = 2*pi, c = pi, rho = 0.99))
```

```
mcauchy7 <- mle2(LLcauchy7, start=list(a = pi, b = pi, c = 0, d = 0, rho = 0.5),
            method = "L-BFGS-B", lower=c(a = -2*pi, b = -2*pi, c = -2*pi, d = -pi, rho = 0.01),
            upper=c(a = 2*pi, b = 2*pi, c = 2*pi, d = pi, rho = 0.99))

mcauchy8 <- mle2(LLcauchy8, start=list(a = pi, b = pi, c = 0, rho = 0.5),
            method = "L-BFGS-B", lower=c(a = -2*pi, b = -2*pi, c = -pi, rho = 0.01),
            upper=c(a = 2*pi, b = 2*pi, c = pi, rho = 0.99))

mcauchy9 <- mle2(LLcauchy9, start=list(a = pi, b = pi, c = 0, d = 0, rho = 0.5),
            method = "L-BFGS-B", lower=c(a = -2*pi, b = -2*pi, c = -2*pi, d = -pi, rho = 0.01),
            upper=c(a = 2*pi, b = 2*pi, c = 2*pi, d = pi, rho = 0.99))

mcauchy10 <- mle2(LLcauchy10, start=list(a = pi, b = pi, c = 0, d = 0, rho = 0.5),
             method = "L-BFGS-B",
             lower=c(a = -2*pi, b = -2*pi, c = -0.1, d = -0.1, rho = 0.01),
             upper=c(a = 2*pi, b = 2*pi, c = 0.1, d = 0.1, rho = 0.99))

mcauchy11 <- mle2(LLcauchy11, start=list(a = pi, b = pi, c = pi, d = 0, e = 0, f = 0, rho = 0.5),
            method = "L-BFGS-B",
            lower=c(a = -2*pi, b = -2*pi, c = -2*pi, d = -0.1, e = -0.1, f = -0.1, rho = 0.01),
            upper=c(a = 2*pi, b = 2*pi, c = 2*pi, d = 0.1, e = 0.1, f = 0.1, rho = 0.99))

### Comparing all models
(comp <- AICctab(mcauchy, mcauchy1, mcauchy2, mcauchy3, mcauchy4, mcauchy5, mcauchy6, mcauchy7,
```

```
        mcauchy8, mcauchy9, mcauchy10, mcauchy11,
        base=T, weights=T, nobs=length(velo)))

# BEST MODEL = no effect model!
# Plotting:
hist(angle, breaks = seq(-pi, pi, length=36), probability = T,
   xlab="Turning angles (rad)", ylab="Probability density", main="")
curve(dwcauchy(x, coef(mcauchy)[1], coef(mcauchy)[2]), -pi, pi, col=2, add=T)

# Plotting likelihodd intervals
mcauchy.prof = profile(mcauchy)
par(mfrow=c(1,2))
plotprofmle2(mcauchy.prof)
par(mfrow=c(1,1))
```
