# Supplementary material for: Effects of Land Cover on the Movement of Frugivorous Birds in a Heterogeneous Landscape
Source: PLoS One. 2016 Jun 3;11(6):e0156688. doi: 10.1371/journal.pone.0156688 (PMC4892584; doi:10.1371/journal.pone.0156688)
Supplement: S2 Text — (PDF) [file pone.0156688.s007.pdf]

S2 Text. Description of the probability distributions used to explain variation in movement data

In order to explain variation in movement speed data, four probability distributions were fit to the data: exponential, Lévy, truncated Lévy, and Weibull distributions.

Exponential distributions are short-tailed and assume that animals perform trajectories within a characteristic spatial scale (individual displacement lengths are centered around a typical value; [1]), so that their path resembles a Brownian motion with normal diffusion properties [2]. Mathematically, the probability density function (*pdf*) *p(x)* of an exponential distribution is described by

$$p(x) = \begin{cases} \lambda e^{-\lambda x}, & x \geq 0 \\ 0, & x < 0, \end{cases}$$

which has only one parameter, λ. This *pdf* is represented in Fig. 1 and is referred as Exponential (λ) in the main text. For more information about exponential distributions, see [3].

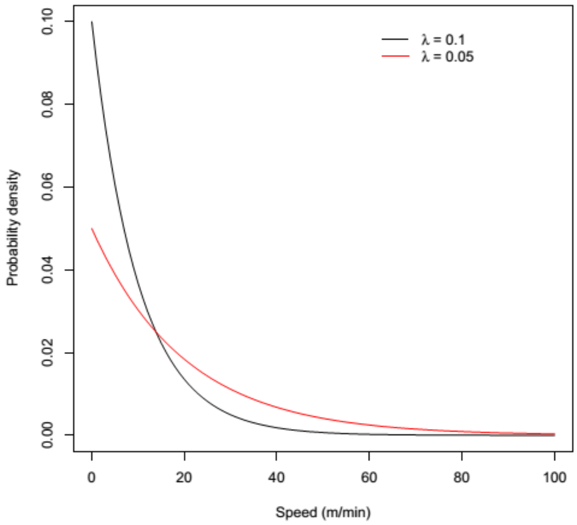

Fig 1. Probability density function for exponential distributions.

Lévy distributions are long-tailed power-law distributions with infinite variance, giving rise to super-diffusive dynamics. They have scale-free properties, which has the consequence of allowing values for displacement lengths and speeds much greater than

the mean values of the distribution. In practice, they can be represented by clusters of short displacements with high tortuosity separated by some very long moves. Truncated Lévy (or bounded Pareto) distributions are modified versions of Lévy distributions, which limit the maximum value of the distribution but still maintain a long-tail and super-diffusion properties over relatively large time extensions [2]. The *pdf* is described by

$$p(x) = \begin{cases} 0, & x < x_{min} \\ \dfrac{\mu - 1}{x_{min}^{1-\mu} - x_{max}^{1-\mu}}\, x^{-\mu}, & x_{min} \leq x < x_{max} \\ 0, & x \geq x_{max}, \end{cases}$$

in which x_{min} is the minimum value (maintained fixed in model fit, see *2. Description of the models used to fit movement data*) and x_{max} is a maximum value (maintained fixed in the model fit, see *2. Description of the models used to fit movement data*), considered only for truncated Lévy distributions. For Lévy distributions, the power-law decay continues as $x \to \infty$. The parameter that governs the behavior of (truncated) Lévy distributions is μ, which typically varies between 1 and 3. When $\mu = 3$, the distribution resembles a Brownian motion, with very few large values of average speed; as $\mu \to 1^+$, the probability of such large value increases. This is shown in Fig. 2 for $x_{min} = 1$ and $x_{max} = 60$, in logarithmic scales, which makes it easier to notice the difference in the probability of large x values among curves. Note that for truncated Lévy distributions no values greater than x_{max} are observed. These distributions are referred as Lévy (μ) and truncated Lévy (μ) in the main text. For more information on (truncated) Lévy distributions, see [1-2].

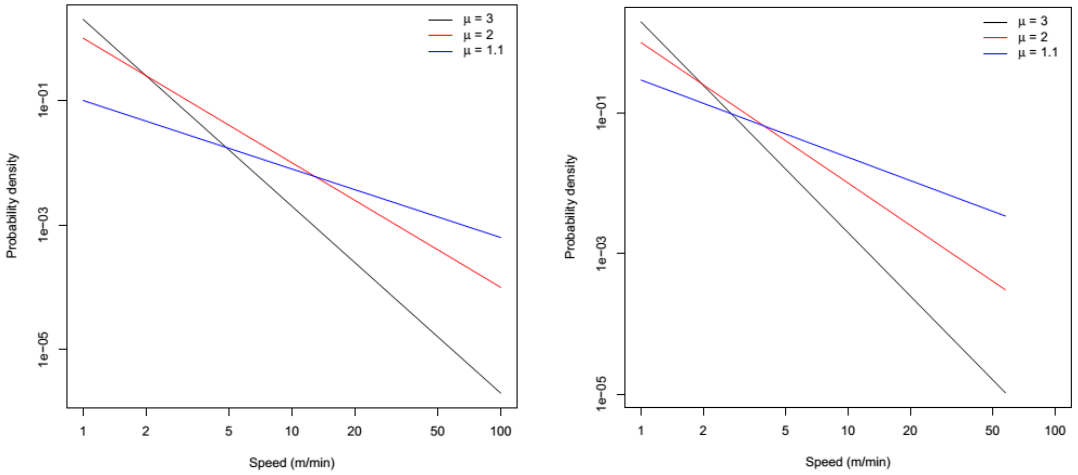

Fig 2. Probability density function for Lévy distribution (left) and truncated Lévy distribution (right).

Weibull distributions are more flexible and can present short- or heavy-tails, or even a non-zero mode, depending on their parameters, which can represent differences in behavior or responses to environmental gradients. Mathematically, their *pdf* are represented by

$$p(x) = \begin{cases} \frac{k}{\lambda}\left(\frac{x}{\lambda}\right)^{k-1} e^{-(x/\lambda)^k}, & x \geq 0 \\ 0, & x < 0. \end{cases}$$

Weibull distributions are described by two parameters: the shape parameter, *k*, that may drastically alter the behavior of the distribution, and the scale parameter, λ. Variations in the distribution due to different values of these two parameters are shown in Fig. 3. This distribution is referred in the main text as Weibull (*k*, λ). For more information on Weibull distributions, see [3].

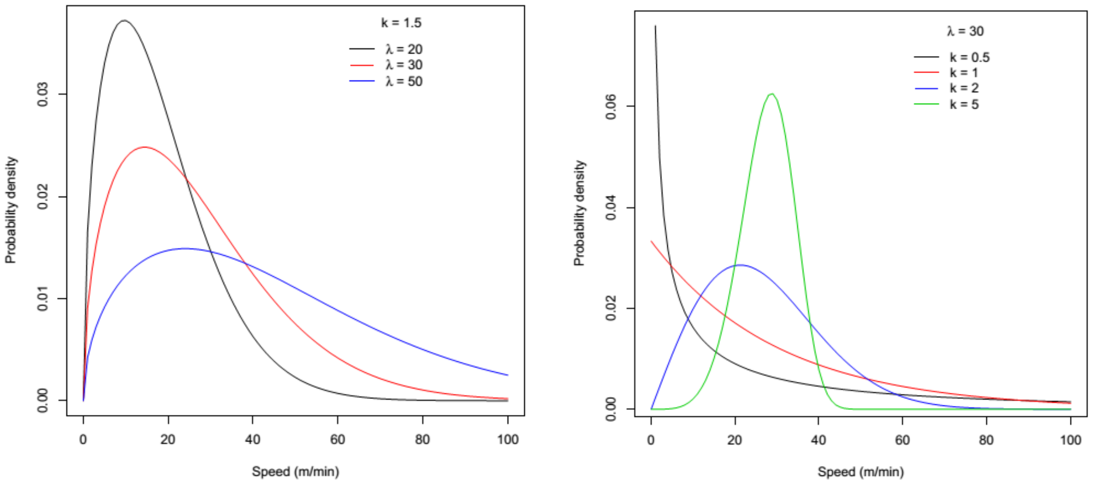

Fig 3. Probability density function for Weibull distribution. On the left, only the scale parameter λ is varied; on the right, we vary only the shape parameter *k*.

For explaining variation in turning angles, we used a wrapped Cauchy distribution. This distribution is suitable for describing angles, since they are bounded between –π and π rad. The *pdf* for wrapped Cauchy distribution is defined mathematically as

$$p(\theta) = \frac{1}{2\pi}\frac{1-\rho^2}{1+\rho^2-2\rho\cos(\theta-\mu)}, \quad \rho \,\epsilon\, [0,1], \quad \theta \,\epsilon\, [0, 2\pi].$$

In the above function, θ is the angle, μ is the peak position of the "unwrapped" Cauchy distribution, that represents the most probable value, and ρ is the scale factor, which describes the variation around μ. The wrapped Cauchy distribution is referred to in the main text as Wrapped Cauchy (π, ρ) and is shown in Fig. 4. For more information about wrapped Cauchy distributions, see [4-5].

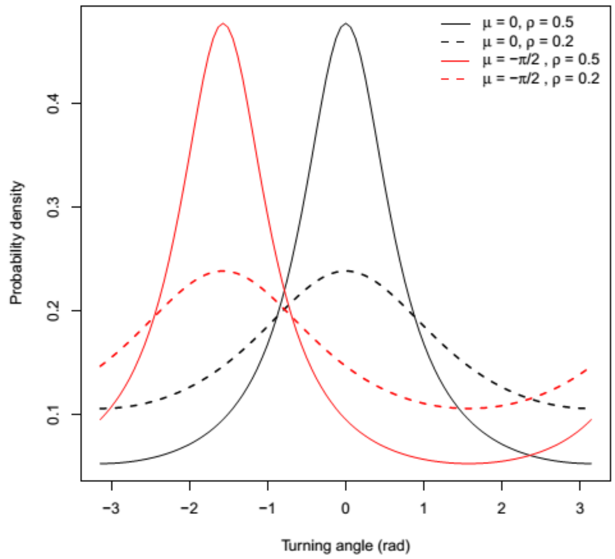

Fig 4. Probability density function for wrapped Cauchy distribution.

References

1. Newman M. Power laws, Pareto distributions and Zipf's law. Contemporary Physics. 2005; 46: 323–351. doi:10.1080/00107510500052444

2. Viswanathan GM, da Luz, Raposo EP, Stanley HE. The Physics of Foraging: an Introduction to Random Searches and Biological Encounters. 1st ed. Cambridge: Cambridge University Press; 2011.

3. Bolker BM. Ecological Models and Data in R. 1st ed. Princeton: Princeton University Press; 2008.

4. Morales JM, Haydon DT, Frair J, Holsinger KE, Fryxell JM. Extracting more out of relocation data: building movement models as mixtures of random walks. Ecology. 2004; 85: 2436–2445. doi: 10.1890/03-0269

5. Bartumeus F, Catalan J, Viswanathan GM, Raposo EP, da Luz MGE. The influence of turning angles on the success of non-oriented animal searches. J Theor Biol. 2008;252: 43–55.
