# Supplementary material for: Effects of Land Cover on the Movement of Frugivorous Birds in a Heterogeneous Landscape
Source: PLoS One. 2016 Jun 3;11(6):e0156688. doi: 10.1371/journal.pone.0156688 (PMC4892584; doi:10.1371/journal.pone.0156688)
Supplement: S3 Text — (PDF) [file pone.0156688.s008.pdf]

S3 Text.Description of the models used to fit movement data.

For fitting exponential and Weibull distributions to average speed data and wrapped Cauchy distributions to turning angles data, we built models considering landscape variables (land cover class, binary cover class, distance to forest edges), sex, and species as covariates, as well as a no effect model (without covariates). In all Weibull models (except the no effect model) as well as in the exponential models that considered distance edges, we used a log link function to ensure that the estimated parameters were positive.

For (truncated) Lévy distributions, we considered only the no effect models, since the great variance in these distributions may already account for variation in real data. Also, we estimated only μ, the power-law exponent. The lower cutoff, x_{min}, was defined as the lowest observed value of average speed. For the truncated Lévy, the higher cutoff, x_{max}, was maintained fixed as the highest observed value of speed, plus 10m/min, given the 10 m of measurement error in the field (see *Methods* section in the main text).

For fitting the models, all categorical variables were transformed in sets of dummy variables. The compared models are shown below. To fit the (truncated) Lévy and wrapped Cauchy models to data and maintain the estimated parameters within a meaningful interval, we constrained the estimation using method "L-BFGS-B" of the mle2 function (package *bbmle*).

Exponential models

$Y \sim Exponential\ (\lambda)$

$M0\colon \lambda = constant$

$M1\colon \lambda = a * binary\ cover\ classes\ (forest) + b * binary\ cover\ classes\ (non\text{-}forest)$

$M2\colon \lambda = a * land\ cover\ classes\ (forest) + b * land\ cover\ classes\ (matrix)$
$\quad + c * land\ cover\ classes\ (urban)$

$M3\colon \lambda = a * species\ (T.leucomelas) + b * species\ (T.rufiventris)$

$M4\colon \lambda = a * sex\ (male) + b * sex\ (female)$

$M5\colon \ln(\lambda) = a + b * distance\ to\ edge$

$M6\colon \lambda = a * sex\ (male)|binary\ cover\ class\ (forest)$
$\quad + b * sex\ (female)|binary\ cover\ class\ (forest)$
$\quad + c * binarycoverclass(non\text{-}forest)$

$M7: \lambda = a * sex\ (male) | binary\ cover\ class\ (forest)$

$\quad + \ b * sex\ (female) | binary\ cover\ class\ (forest)$

$\quad + c * binary\ cover\ class\ (non\text{-}forest) + \ d * binary\ cover\ class (urban)$

$M8: \lambda = a * species\ (T. leucomelas) | binary\ cover\ class\ (forest)$

$\quad + \ b * species\ (T. rufiventris) | binary\ cover\ class\ (forest)$

$\quad + c * binary\ cover\ class\ (non\text{-}forest)$

$M9: \lambda = a * species\ (T. leucomelas) | binary\ cover\ class\ (forest)$

$\quad + \ b * species\ (T. rufiventris) | binary\ cover\ class\ (forest)$

$\quad + c * binary\ cover\ class\ (non\text{-}forest) + \ d * binary\ cover\ class (urban)$

$M10: ln\ (\lambda) = \ a * binary\ cover\ classes\ (forest)$

$\quad + b * binary\ cover\ classes\ (non\text{-}forest)$

$\quad + c * distance\ to\ edge\ (in\ forest)$

$\quad + \ d * distance\ to\ edge\ (in\ non\text{-}forests)$

$M11: \ln(\lambda) = \ a * binarycoverclasses(forest) + b * binarycoverclasses(matrix) + c$

$\quad * binarycoverclasses(urban) + d * distancetoedge(inforest) + \ e$

$\quad * distancetoedge(inmatrix) + \ f * distancetoedge\ (inurban)$

Lévy model

$Y \sim Lévy(\mu)$

Truncated Lévy model

$Y \sim truncated\ Lévy(\mu)$

Weibull models

$Y \sim Weibull\ (k, \lambda)$

$M0: k = constant$

$M1: \ln(k) = a * binary\ cover\ classes\ (forest)$

$\quad + b * binary\ cover\ classes\ (non\text{-}forest)$

$M2: \ln(k) = a * land\ cover\ classes\ (forest) + b * land\ cover\ classes\ (matrix)$

$\quad + c * land\ cover\ classes\ (urban)$

$M3: \ln(k) = a * species\ (T. leucomelas) + b * species\ (T. rufiventris)$

$M4: \ln(k) = a * sex\ (male) + b * sex\ (female)$

$M5: \ln(k) = a + b * distance\ to\ edge$

$$M6: \ln(k) = a * sex\ (male)|binary\ cover\ class\ (forest)$$
$$+\ b * sex\ (female)|binary\ cover\ class\ (forest)$$
$$+ c * binarycoverclass(non\text{-}forest)$$

$$M7: \ln(k) = a * sex\ (male)|binary\ cover\ class\ (forest)$$
$$+\ b * sex\ (female)|binary\ cover\ class\ (forest)$$
$$+ c * binary\ cover\ class\ (non\text{-}forest) +\ d * binary\ cover\ class(urban)$$

$$M8: \ln(k) = a * species\ (T.leucomelas)|binary\ cover\ class\ (forest)$$
$$+ b * species\ (T.rufiventris)|binary\ cover\ class\ (forest)$$
$$+ c * binary\ cover\ class\ (non\text{-}forest)$$

$$M8: ln(k) = a * species\ (T.leucomelas)|binary\ cover\ class\ (forest)$$
$$+ b * species\ (T.rufiventris)|binary\ cover\ class\ (forest)$$
$$+ c * binary\ cover\ class\ (non\text{-}forest) +\ d * binary\ cover\ class(urban)$$

$$M10: \ln(k) =\ a * binary\ cover\ classes\ (forest)$$
$$+ b * binary\ cover\ classes\ (non\text{-}forest)$$
$$+ c * distance\ to\ edge\ (in\ forest)$$
$$+\ d * distance\ to\ edge\ (in\ non\text{-}forests)$$

$$M11: \ln(k) =\ a * binarycoverclasses(forest) + b * binarycoverclasses(matrix) + c$$
$$* binarycoverclasses(urban) + d * distancetoedge(inforest) +\ e$$
$$* distancetoedge(inmatrix) +\ f * distancetoedge\ (inurban)$$

Wrapped Cauchy models

$$Y \sim wrapCauchy\ (\mu, \rho)$$

$$M0: \mu = constant$$

$$M1: \mu = a * binary\ cover\ classes\ (forest) + b * binary\ cover\ classes\ (non\text{-}forest)$$

$$M2: \mu = a * land\ cover\ classes\ (forest) + b * land\ cover\ classes\ (matrix)$$
$$+ c * land\ cover\ classes\ (urban)$$

$$M3: \mu = a * species\ (T.leucomelas) + b * species\ (T.rufiventris)$$

$$M4: \mu = a * sex\ (male) + b * sex\ (female)$$

$$M5: \mu = a + b * distance\ to\ edge$$

$$M6: \mu = a * sex\ (male)|binary\ cover\ class\ (forest)$$
$$+ b * sex\ (female)|binary\ cover\ class\ (forest)$$
$$+ c * binarycoverclass(non\text{-}forest)$$

$$M7: \mu = a * sex\ (male) | binary\ cover\ class\ (forest)$$
$$+\ b * sex\ (female) | binary\ cover\ class\ (forest)$$
$$+ c * binary\ cover\ class\ (non\text{-}forest) +\ d * binary\ cover\ class (urban)$$
$$M8: \mu = a * species\ (T. leucomelas) | binary\ cover\ class\ (forest)$$
$$+\ b * species\ (T. rufiventris) | binary\ cover\ class\ (forest)$$
$$+ c * binary\ cover\ class\ (non\text{-}forest)$$
$$M9: \mu = a * species\ (T. leucomelas) | binary\ cover\ class\ (forest)$$
$$+\ b * species\ (T. rufiventris) | binary\ cover\ class\ (forest)$$
$$+ c * binary\ cover\ class\ (non\text{-}forest) +\ d * binary\ cover\ class (urban)$$
$$M10: \mu =\ a * binary\ cover\ classes\ (forest) + b * binary\ cover\ classes\ (non\text{-}forest)$$
$$+ c * distance\ to\ edge\ (in\ forest)$$
$$+\ d * distance\ to\ edge\ (in\ non\text{-}forests)$$
$$M11: \mu =\ a * binarycoverclasses(forest) + b * binarycoverclasses(matrix) \quad + c$$
$$* binarycoverclasses(urban) + d * distancetoedge(inforest) +\ e$$
$$* distancetoedge(inmatrix) +\ f * distancetoedge\ (inurban)$$
