# Supplementary material for: Effects of Land Cover on the Movement of Frugivorous Birds in a Heterogeneous Landscape
Source: PLoS One. 2016 Jun 3;11(6):e0156688. doi: 10.1371/journal.pone.0156688 (PMC4892584; doi:10.1371/journal.pone.0156688)
Supplement: S4 Text — (PDF) [file pone.0156688.s009.pdf]

S4 Text. Details of movement data fit.

For average speeds, the most likely model was Exponential M11, which explains variation in speeds according to the interaction between land cover class and distance to edge. The estimates of the parameters for this model are shown below. We also show the values for the likelihood intervals of each parameter, i.e., the interval in which the difference between the negative log-likelihood of all values and the negative log-likelihood of the estimate is smaller than ln(8) ≈ 2.1. Likelihood intervals were calculated using a modified version of the function *profile*, from package *sads*. This is also shown in Fig. 1.

Parameter	Parameter estimate (likelihood interval)	Parameter	Parameter estimate (likelihood interval)
a	1.75 (1.44 – 2.06)	d	-0.0084 (-0.0172 – -0.0010)
b	2.56 (2.90 – 2.82)	e	-0.0089 (-0.0124 – -0.0058)
c	1.93 (1.54 – 2.29)	f	-0.0034 (-0.0091 – 0.0014)

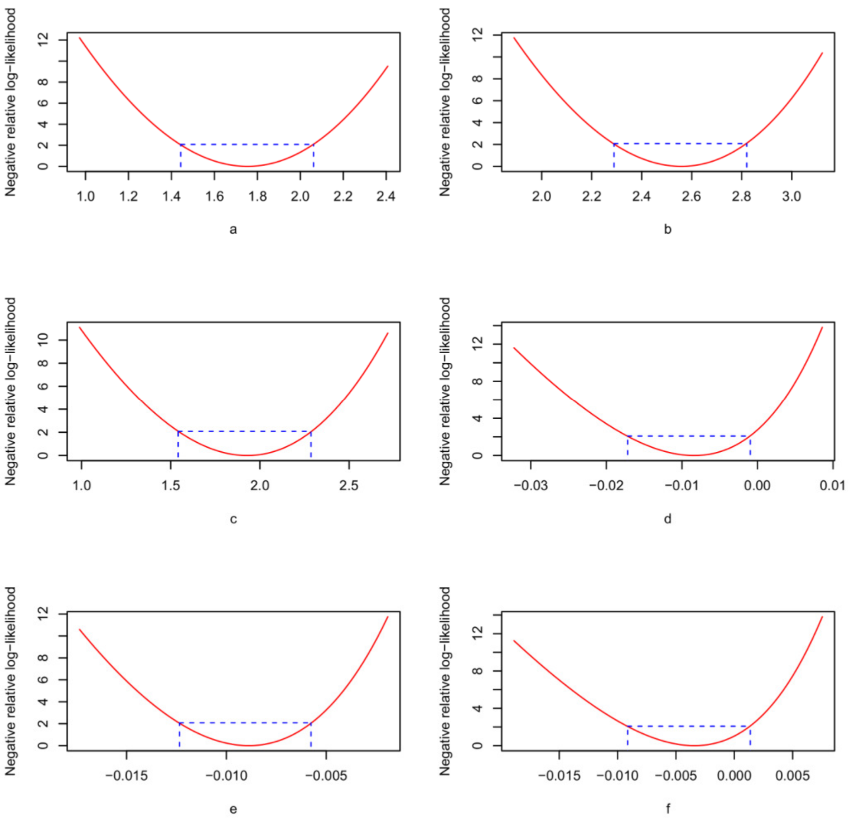

Fig 1. Likelihood intervals for model Exponential M11.

For turning angles, the no effect model (M0) was as likely as many of the other models; since it is simpler, we show the results for it. The estimates and likelihood intervals of the parameters for this model are shown below, as well as in Fig. 2.

Parameter	Parameter estimate (likelihood interval)
μ	3.02 (2.76 – 3.27)
ρ	0.31 (0.22 – 0.40)

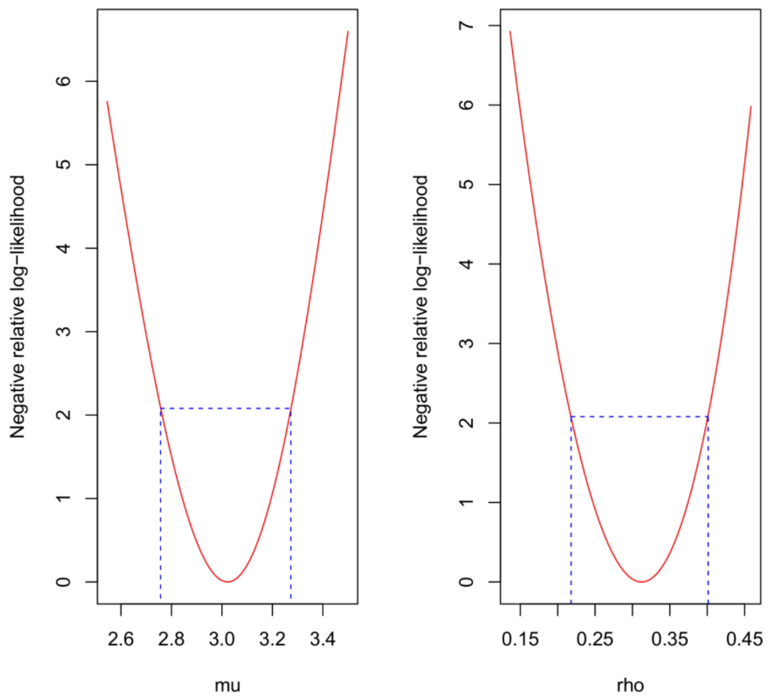

Fig 2. Likelihood intervals for the wrapped Cauchy model, M0.
