# Supplementary material for: Effects of Land Cover on the Movement of Frugivorous Birds in a Heterogeneous Landscape
Source: PLoS One. 2016 Jun 3;11(6):e0156688. doi: 10.1371/journal.pone.0156688 (PMC4892584; doi:10.1371/journal.pone.0156688)
Supplement: S5 Text — (PDF) [file pone.0156688.s010.pdf]

S5 Text. Considerations of individual variation on the effects of land cover on thrush movement.

To assess whether the effects of land cover on the movement of thrushes were influenced by variation in the characteristics and ecology of the sampled individuals, we performed two kinds of tests. First, we tested for individual variation using Kruskal-Wallis and Mann-Whitney tests for movement parameters: average speed and turning angle. Second, we included individuals as random effects in the most plausible model for average speed (Model M11, see Table 1, main text) to evaluate differences in the results. Both approaches are shown below. Although there is some individual variation, the effects of land cover on movement were qualitatively the same once this variation was accounted for.

1) Testing individual variation

In order to test for individual variation, we performed a Kruskal-Wallis test, which had the null hypothesis that individual average speeds (and individual turning angles) are identical populations, i.e., belong to the same Gaussian distribution. We detected variation among individuals for average speeds ($\chi^2 = 24.14$, df = 9, p = 0.0041) but not for turning angles ($\chi^2 = 8.31$, df = 9, p = 0.5032). As there was evidence that individual thrush speeds were non-identical populations, we performed individual-to-individual Mann-Whitney tests to detect which individuals had different average speeds (again, the null hypothesis was that individual speeds came from the same distribution). P values for each pair of individuals' test is shown below, in Table S5-1. There is indeed some difference between individuals (here we adopted a significance level $\alpha = 0.05$).

Table S5-1. P values for Mann-Whitney tests for each pair of individuals. Values < α = 0.05 were considered significant. Numbers in rows and columns represent individuals.

	1	2	3	4	5	6	7	8	9	10
1	-									
2	0.86	-								
3	**0.02**	**0.002**	-							
4	0.07	**0.02**	0.36	-						
5	0.73	0.49	**0.02**	**0.03**	-					
6	0.12	**0.01**	0.74	0.65	0.05	-				
7	0.07	**0.04**	0.10	0.43	0.06	0.35	-			
8	0.82	0.75	**0.01**	**0.03**	0.67	0.08	**0.04**	-		
9	0.72	0.94	0.16	0.19	0.71	0.20	0.31	0.79	-	
10	0.80	0.33	0.02	0.76	0.76	0.06	0.09	0.62	0.60	-

Significant values (at the 0.05 significance level) are shown in bold.

From these analyses, we conclude that there is some variation between individuals, at least for average speeds. The question that follows is: does this variation between individuals alter the results?

2) Inter-individual variation and effects of land cover and edge distance on thrush movement

In order to assess the influence of individual variation on the results, we built hierarchical models, considering individuals as random effects. In this approach, data on all individuals are used to calculate parameters for the pool of individuals; then individual parameters are considered as random samples from this common Gaussian distribution. In this way, each individual has different parameters for the fitted models (individuals are different), but the parameters are not independent (they are related, since individuals are somehow ecologically similar) [1,2].

As hierarchical models are somewhat more complex than non-hierarchical ones, we needed a Bayesian approach to fit them. Models were fit using a Markov Chain Monte Carlo (MCMC) algorithm in the software JAGS (Just Another Gibbs Sampler) [3]. All codes were implemented through R, using R2jags package [4] (codes are available in Supplementary Material S6 Text).

As the aim was to verify if taking individuals into account altered the results, only the most plausible model for average speed was modified (M11, in which speed depended on the interaction of land cover and distance to forest edges; see Table 1, main text). However, the same approach

may be applied to the other models. The new hierarchical model included average speeds as the response variable, following an exponential variation and included the interaction of the fixed effects variables, land cover type and distance to edge, and the individuals as random factors on the model intercept. All parameters were given uninformative priors, so that, besides taking individuals as random effects, all the other parts of the model were equivalent to the non-hierarchical model (M11). For model fitting, we generated three 30 thousand long chains and discarded the first 5000 values of each chain.

When we look for variation among individuals included in the model, we have the same qualitative result for speeds as when we run the model without the random effects (compare Fig. S5-1 below with Fig. 4 in the main text): there is an effect of land cover, and the speed increases as distance to forest edge increases. As the results were qualitatively the same, we conclude that, although there is some variation between individuals, this variation does not alter the general results.

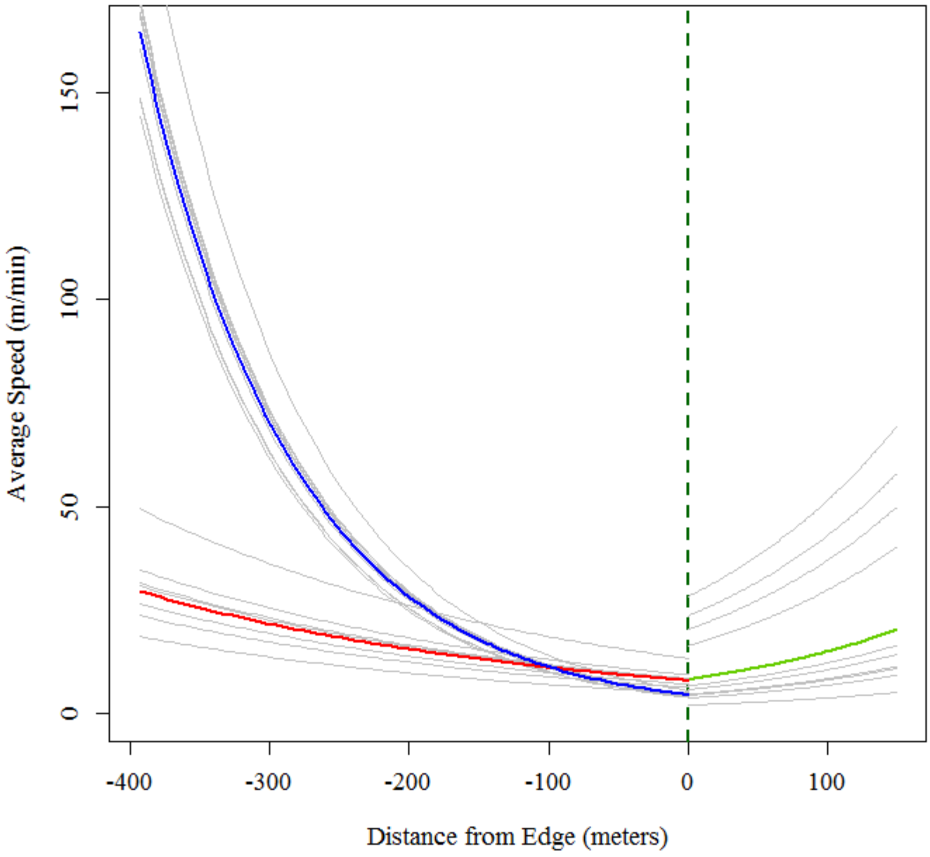

Fig. S5-1. Average speeds of thrushes as a function of distances from edge, considering land cover classes and individuals as random effects. The dashed line represents the forest edges; green (forest), blue (open matrix: pastures and crops) and red (urban areas) lines represent the expected values for the mean speeds of the pool of individuals. Grey lines represent each individual

response to land cover. As in Fig. 4 (main text), speeds increase as individuals move away from forest edges.

References

[1] Gelman A, Hill J. Data Analysis Using Regression and Multilevel/Hierarchical Models. New York: Cambridge University Press; 2009.

[2] Kéry M, Schaub M. Bayesian Population Analysis using WinBUGS: a hierarchical perspective. Waltham: Academic Press; 1012.

[3] JAGS website: http://mcmc-jags.sourceforge.net/.

[4] Yu-Sung Su, Masanao Yajima. R2jags: Using R to Run 'JAGS'. R package version 0.5-7. 2015; http://CRAN.R-project.org/package=R2jags.
