# Supplementary material for: Effects of Land Cover on the Movement of Frugivorous Birds in a Heterogeneous Landscape
Source: PLoS One. 2016 Jun 3;11(6):e0156688. doi: 10.1371/journal.pone.0156688 (PMC4892584; doi:10.1371/journal.pone.0156688)
Supplement: S6 Text — (PDF) [file pone.0156688.s011.pdf]

S6 Text. Hierarchical model script including individual variation.

```
############################################################
#
# S6 Text. Hierarchical model script including individual variation.
#
###########################################################
#
# Movement analysis for thrushes in fragmented landscapes
# Comparing MLE and Bayesian models - best models
#
# Natalia Stefanini Silveira - nat.stefanini at gmail.com
# Bernardo Niebuhr - bernardo_brandaum at yahoo.com.br
#
# Reference: Silveira et al. 2016.
# Effects of land cover on the movement of frugivorous
# birds in a heterogeneous landscape. PLoS One.
#
# No rights reserved - feel free to modify and share
###########################################################

if(!require(adehabitatLT)) install.packages("adehabitatLT", dep=T);
library(adehabitatLT)
if(!require(bbmle)) install.packages("bbmle", dep=T); library(bbmle)
if(!require(circular)) install.packages("circular", dep=T); library(circular)
if(!require(R2jags)) install.packages("R2jags", dep=T); library(R2jags)

###################
```

```
# Loading and organizing data
rm(list=ls())

# Changing working directory
#setwd("")

# Reading data
data <- read.table("dados_final.csv", sep=",",dec=".", header=TRUE)

head(data)
str(data)
names(data)=c("nome", "sexo", "data","hora", "especie", "xestimate", "yestimate",
        "id_poligono", "classe", "classe_bin", "peso", "sitio", "dist_borda")

data$prox_borda <- ifelse(data$dist_borda > 0, data$dist_borda, -data$dist_borda)

# Transforming data into ltraj class
data$data<-as.character(data$data)
data$hora<-as.character(data$hora)

da <- paste(data$data, data$hora)
da

bursts <- paste(data$nome, data$data, sep="_")

da1 <- as.POSIXct(strptime(da, format="%Y-%m-%d %H:%M"))
```

```
path <- as.ltraj(xy = data[,c("xestimate", "yestimate")], date = da1, id = data$nome,
           burst=bursts, typeII=T, infolocs=data[,c("sexo", "especie", "id_poligono",
                                                    "classe", "classe_bin",
                                                    "peso", "sitio", "dist_borda",
                                                    "prox_borda")])

# 3 classes
path.df$class3 <- as.character(path.df$classe_bin)
path.df$class3[path.df$classe == "res"] <- "URB"
path.df$class3 <- as.factor(path.df$class3)

# Deleting fixes with dt > 2h = 7200s
path.df <- path.df[!is.na(path.df$dist),]
path.df <- path.df[path.df$dt < 7200,]

# Separating dependent variables
dist <- path.df$dist
time <- path.df$dt[!is.na(path.df$dt)]

# Average speed
velo <- dist/time*60
# Turning angles
angle <- path.df$rel.angle[!is.na(path.df$rel.angle)]

# Separating independent variables
# for Y = Mean speed
class.bin.v = path.df$classe_bin
```

```
species.v = as.factor(path.df$especie)
site.v = as.factor(path.df$sitio)
sex.v = as.factor(path.df$sexo)
class3.v = path.df$class3
distedge.v = path.df$dist_borda
proxedge.v = path.df$prox_borda
id <- path.df$id

### Defining models
# Exponential models

n <- length(velo)
ids <- unique(id)
nid <- length(unique)

# function to search and replace
# took from flodel at GitHUb
# https://gist.github.com/flodel/4470993
decode <- function(x, search, replace, default = NULL) {

  # build a nested ifelse function by recursion
  decode.fun <- function(search, replace, default = NULL)
    if (length(search) == 0L) {
      function(x) if (is.null(default)) x else rep(default, length(x))
    } else {
      function(x) ifelse(x == search[1L], replace[1L],
                         decode.fun(tail(search,  -1L),
```

```
                                tail(replace, -1L),
                                default)(x))
    }

  return(decode.fun(search, replace, default)(x))
}

id2 <- as.factor(decode(id, search = ids, replace = 1:length(ids)))
ids2 <- unique(id2)

######################################
# This is an exponential model, considering both the effects of
# edge and land cover class
# Also considering a individuals as a random intercept
# (partial pooling for intercepts)

# Save BUGS description of the model to working directory
sink("model_best.txt")
cat("
    model {


    # Priors
    for(i in 1:nind) {
    for(j in 1:nclass) {
    alpha[i,j] ~ dnorm(mu[j], tau[j]) # random intercepts
    }
    }
```

```
    for(i in 1:nclass) {
    beta[i] ~ dnorm(0, 1.0E-06) # fixed slopes

    mu[i] ~ dnorm(0, 1.0E-06) # hyperparameter priors
    tau[i] <- 1/(sd[i] * sd[i])
    sd[i] ~ dunif(0, 100)
    }

    # Likelihood
    for(i in 1:nobs){
    velo[i] ~ dexp(lambda[i])
    lambda[i] <- exp(alpha[ind[i],cover[i]] + beta[cover[i]]*edge[i])
    }
    }
    ",fill=TRUE)
sink()

# Package all the stuff to be handed over to WinBUGS
# Bundle data
win.data <- list(velo = velo, nobs = length(velo),
            cover = as.numeric(class3.v),
            nclass = length(levels(class3.v)),
            ind = as.numeric(id), nind = length(levels(id)),
            edge = proxedge.v)

# Function to generate starting values
```

```
glm.out <- glm(velo ~ proxedge.v, family=Gamma(link=log))

coef(glm.out)


alfa <- matrix(coef(glm.out)[1], nrow = length(levels(id)), ncol =
length(levels(class3.v)))

inits <- function() {

  list(alpha = alfa,

       beta = rep(coef(glm.out)[2], length(levels(class3.v))))

}


# Parameters to be monitored (= to estimate)

params <- c("alpha", "beta", "mu", "sd")


# MCMC settings

nc <- 3           # Number of chains

ni <- 30000    # Number of draws from posterior (for each chain)

nb <- 5000                # Number of draws to discard as burn-in

nt <- 1           # Thinning rate


# Start Gibbs sampler: Run model in WinBUGS and save results in object called out

out_best <- jags(data = win.data, inits = inits, parameters.to.save = params,

              model.file = "model_best.txt", n.thin = nt, n.chains = nc, n.burnin = nb, n.iter
= ni,

              DIC = F, working.directory = getwd())


# Results:

print(out_best, intervals=c(0.025, 0.5, 0.975))

out_best$BUGSoutput$summary
```

```
plot(out_best)
```
